# An anti-CEA affibody showing high-definition staining in human pancreatic cancer tissue sections and selective tumor targeting *in vivo*

**DOI:** 10.1016/j.tranon.2025.102512

**Published:** 2025-08-28

**Authors:** Johan Nilvebrant, Carlos Fernández Moro, Eleftherios Papalanis, Masih Ostad Novin, Haozhong Ding, Ruonan Li, Maryam Oroujeni, Arun Selvam, Béla Bozóky, Torbjörn Gräslund, Timea Szekerczes, Tatiana Sandalova, Hugh Salter, Adnane Achour, Vladimir Tolmachev, Mikael Björnstedt, Per-Åke Nygren

**Affiliations:** a Department of Protein Science, AlbaNova University Center, KTH Royal Institute of Technology, SE-144 21 Stockholm, Sweden; b Department of Clinical Pathology and Cancer Diagnostics, Karolinska University Hospital F46, SE-141 86, Stockholm, Sweden; c Division of Pathology, Department of Laboratory Medicine, Karolinska Institute, SE-141 52 Huddinge, Sweden; d Department of Immunology, Genetics and Pathology, Uppsala University, SE-751 85, Uppsala, Sweden; e Science for Life Laboratory, Department of Medicine, Karolinska Institute, SE-171 65 Solna, Sweden; f Division of Infectious Diseases, Karolinska University Hospital, SE-14186 Stockholm, Sweden; g Science for Life Laboratories, Karolinska Institute, SE-171 65 Solna, Sweden

**Keywords:** Affibody, Affinity, Phage display, Carcinoembryonic antigen, CEACAM5, CEA, HRP, Immunohistochemistry, IHC, Pancreatic cancer, Adenocarcinoma, II-7, CEA31, Clinical diagnostics, Xenograft, *In vivo* imaging

## Abstract

We report development and characterization of small non-immunoglobulin affibody affinity proteins directed to the highly glycosylated human carcinoembryonic antigen-related adhesion molecule 5 (CEACAM5, CEA), and their use in immunohistochemical (IHC) analyses of human pancreatic cancer samples and for *in vivo* tumor imaging. A total of nineteen unique anti-CEA affibodies were identified from large phage display libraries constructed using combinatorial protein engineering of a small 58 amino acid three-helix bundle protein domain. Molecular modeling suggested that all enriched clones share a binding surface with several clustered tryptophan residues interacting with a hydrophobic patch in the N1 domain of CEA centered around a phenylalanine residue. One variant, designated as C9, exhibited the highest affinity in biosensor analyses and was reformatted into a 15 kDa homodimer expressed in *Escherichia coli*. The biotinylated form, C9-C9-Bio, was evaluated for its IHC performance on matched frozen and formalin-fixed, paraffin-embedded (FFPE) sections of human pancreatic cancer samples (*n* = 7). Compared to clinical-grade monoclonal antibodies II-7 and CEA31, as well as a polyclonal reagent, C9-C9-Bio demonstrated highly sensitive CEA detection with minimal background staining. Statistical analyses including intraclass correlation and Bland-Altman assessments revealed excellent agreement between C9-C9-Bio and the two monoclonal antibodies in FFPE tissue samples. Further, a ^99m^Tc[Tc]-labeled C9-C9 construct showed CEA-dependent binding to human cancer cell lines *in vitro*, and selectively bound to CEA-expressing BxPC3 xenografts in mice when investigated as a tracer for *in vivo* imaging, allowing for a visualization of tumors after four hours. In summary, these findings highlight the potential use of the easily produced CEA-binding C9 affibody for various clinical applications, including IHC and medical imaging, and as a targeting moiety for directing various therapeutic modalities to CEA-expressing tumors.

## Highlights

• Development of small anti-CEA affibody affinity proteins.
• High-definition IHC staining of human pancreatic cancer samples.
• Rapid *in vivo* imaging of CEA-expressing xenografts in mice.

## Introduction

CEA is a well-established marker for adenocarcinomas, including colonic and pancreatic carcinomas [5,26,44]. CEA is a highly glycosylated glycosylphosphatidyl-inositol (GPI)-anchored protein predominantly expressed on the surface of cells within the gastrointestinal tract during fetal development, where it plays a critical role in cell adhesion. Postnatally, CEA expression becomes highly restricted; however, it is frequently re-expressed and overexpressed in multiple adenocarcinomas including those originating in the colon, pancreas, stomach, and lung [21,49]. This re-expression renders CEA a widely utilized diagnostic marker, and a potential therapeutic target in clinical oncology. Assessment of CEA expression in surgical samples is commonly used by clinical pathologists for monitoring of cancer spread and as a guide for reductive surgery. To this end, frozen or FFPE patient biopsy samples are routinely analysed by IHC using anti-CEA mouse monoclonal antibodies. The antibody must display high affinity, and high CEA specificity with minimal background or false staining. Several well-performing anti-CEA mouse monoclonal antibodies (*e.g*. II-7, CEA31), produced using hybridoma technology, have been certified for routine clinical diagnostic use [28]. These widely used antibody reagents are combined with specific secondary reagents to increase assay sensitivity, including linked horseradish peroxidase (HRP) polymers, comprising hundreds of HRP enzyme molecules amplifying the staining intensity by greatly enhancing HRP substrate 3,3′-Diaminobenzidine (DAB) conversion.

Given its clinical significance, there have been concerted efforts to develop recombinant anti-CEA antibodies, either by cloning immunoglobulin genes from a mouse hybridoma expressing a well-performing anti-CEA antibody (*e.g.* clone T84.66) [12,23,50], via phage display technology, *e.g*. the single-chain variable fragment (scFv) clones MFE-23 and F4 [11,36] or via development of the nanobody NbCEA5 [47]. Such efforts are well in line with the current view that affinity reagents used in research and clinical diagnostics should be defined by their sequences and available as reproducible recombinant protein reagents [4,9].

Today, affinity proteins also of non-immunoglobulin origin can be developed, where stable protein domains are utilized as scaffolds for combinatorial engineering and directed evolution efforts to generate variants with desired binding specificities [6,25,37,39,40]*.*

One of the first described and also most well-characterized classes of such non-Ig affinity proteins are the affibody affinity proteins, based on a merely 58 amino acid (*ca.* 6.5 kDa) three-helix bundle domain scaffold [30,32]. Numerous affibodies directed to targets of different sizes and structures have been selected from combinatorial libraries displayed on *e.g.* phages, bacteria or yeast [8,43]. Several affibody variants are in pre-clinical development or are currently undergoing clinical trials for *in vivo* molecular imaging or therapy applications, including targets such as HER2 (ABY-025, GE-226), complement factor C5 (RLYB116) and IL-17A (Izokibep) [3,17,42].

In this study, we describe the development and characterization of novel CEA-binding affibody affinity proteins. Several CEA-binding variants were identified from *in vitro* selection using affibody phage display libraries, and the most promising variant, denoted C9, was extensively characterized with respect to binding affinity, thermal stability and mutational analysis of the paratope. A putative binding site on CEA was identified via competitive binding analyses and AlphaFold-based molecular modelling. A high-affinity head-to-tail dimer of the C9 affibody was designed and compared with clinically validated monoclonal antibodies in immunohistochemical detection of human CEA in frozen as well as FFPE pancreatic cancer patient tissue samples. Furthermore, an *in vivo* study in mice showed that a ^99m^Tc-labeled dimeric C9-C9 construct rapidly accumulated in a CEA-expressing tumor xenograft and sensitively and specifically labeled the engrafted cells.

## Materials and methods

### Affibody libraries

The affibody phage display libraries used for selections within this study have been constructed earlier [18], and are based on *lac* promoter controlled phagemids containing an outer membrane protein A (Omp A) signal peptide. These libraries were designed for phage display of encoded affibody library members as in-frame fusions to an albumin-binding domain (ABD), an amber stop codon and either a full-length (Lib-1) or a truncated version (aa 249–406) (Lib-2) of non-lytic filamentous phage M13 phage protein 3. Library members in Lib-1 also contain a trypsin sensitive GARRAG amino acid sequence [45] in front of p3 to facilitate proteolytic elution of phage during selections. Both libraries were made by randomization of 14 positions (pos. 9, 10, 11, 13, 14, 17, 18, 24, 25, 27, 28, 31, 32 and 35) in helices 1 and 2 of the affibody scaffold using trinucleotide codon technology. Codons for all 20 amino acids except Cys and Pro were allowed at an even distribution, except for position 31 where codons för four amino acids were allowed; 60 % Ile and 10 % each of His, Tyr, Lys and Asp. The estimated complexities of Lib-1 and Lib-2 are 4.7 × 10^9^ and 3.0 × 10^10^, respectively.

### Phage stock preparations

Phage stocks for first round selections were produced from *E. coli* ER2738 library cell stocks using standard procedures as previously described [18,19] using either KM13 helper phage (Creative Biolabs, New York, USA) for Lib-1 or M13KO7 helper phage (New England Biolabs, Massachusetts, USA) for Lib-2, where KM13 helper phage encode a trypsin sensitive version of p3. *E. coli* XL-1 Blue cells were used for phage stock production in successive cycles and for phage-ELISA experiments.

### Selection, phage-ELISA screening and DNA sequencing

Selections were performed essentially as previously described [18,19] Briefly, the affibody phage libraries were subjected to four rounds of panning to biotinylated CEA-Avitag-His_6_ protein (CE5-H82EO, Acro Biosystems, Delaware, USA) coupled to streptavidin (SA)-coated paramagnetic beads (M280-SA, ThermoFisher, Massachusetts, USA). In successive cycles, the target protein concentration was decreased from 200 nM in cycle 1 to 25 nM in cycle 4, and the number of washes increased from one (cycle 1) to ten (cycle 4). For both libraries, phage-target protein complexes were eluted from the beads using either low pH (HAc, pH 2.8) or trypsin (0.25 mg/ml, 30 min) in parallel tracks. After the fourth cycle, individual clones were screened for target binding using phage-enzyme-linked immunoadsorbent assay (ELISA) with individually prepared monoclonal phage stocks. Microtiter plates were coated with human serum albumin (HSA) (positive control, binding to an ABD domain present on all affibody-displaying phage particles), CEA, SA as a negative control and (bovine serum albumin) BSA as an additional negative control. A mouse monoclonal anti-M13- HRP conjugate (Merck, Steinheim, Germany) was used together with a 3,3´, 5,5’ tetramethylbenzidine (TMB) substrate for detection. Reactions were stopped by addition of 2 M H_2_SO_4_ and plates were read at 450 nm using a plate-reader (Clariostar, BMG Ortenberg, Germany). The affibody gene inserts in twenty-four phage-ELISA positive clones per track were sent for DNA sequencing (Microsynth Seqlab, Göttingen, Germany).

### Affibody cloning, protein production and purification

Affibody gene inserts of selected clones were amplified by polymerase chain reaction (PCR) and inserted via In-Fusion cloning from Takara (Shiga, Japan) into an in-house phage T7 promoter-based expression vector for intracellular production of hexahistidyl (His_6_)-affibody-ABD fusion proteins in *E. coli* BL21(DE3) cells. Protein production followed standard procedures including the induction of overnight cultures grown at 37 °C with 0.5 mM isopropyl β-d-1-thiogalactopyranoside (IPTG). Proteins were purified from either lysed or sonicated samples by immobilized metal ion chromatography (IMAC) under denaturing or non-denaturing conditions, including elution with 300 mM imidazole. Based on the anti-CEA affibody clones C9 and C11, second-generation constructs including C9-His_6_, C9-C9-His_6_, C11-C11-His_6_, His_6_-C9-Cys, His_6_-C9-C9-Cys and His_6_-C11-C11-Cys were assembled via In-Fusion cloning and produced using similar protocols. Further, a set of 14 individual C9 variants in which the randomized positions 9, 10, 11, 13, 14, 17, 18, 24, 25, 27, 28, 31, 32 and 35 were mutated to alanine, were constructed via PCR mutagenesis in the format C9_Ala mut_-His_6_. Sequences of all constructs were verified by DNA sequencing (Eurofins, Ebersberg, Germany) and proteins produced and purified as described above. Circular dichroism spectroscopy analyses were performed on an Applied Photophysics instrument (Leatherhead, United Kingdom) on the wild type C9 affibody and alanine variants where binding was strongly affected (W9A, W10A, F11A, W13A) to investigate the secondary structure contents and thermal stability. The C9-C9-EYEC affibody construct used in the biodistribution study was produced as a His_6_-tobacco etch virus (TEV) linker (ENLYFQM)-C9-C9-EYEC fusion protein. Following IMAC purification, the His_6_ tag was cleaved off using a His_6_-tagged TEV protease (50 mM protease in Tris–HCl, 0.5 mM ethylenediaminetetraacetic acid (EDTA), 1 mM dithiothreitol (DTT) for 60 min in 30 °C). Following protease cleavage, His_6_-tag containing proteins were removed by IMAC, and the flowthrough containing the C9-C9-EYEC construct was collected.

### MFE-23 scFv cloning, production and purification

The sequence of the anti-CEA scFv fragment MFE-23 was obtained from the Protein Structure Data Bank (PDB) entry 1QOK.pdb. An expression construct in the vector pET22b(+) including a T7 promoter, PelB signal peptide, C-terminal His_6_ tag was obtained from GenScript Biotech BV (Netherlands). *E. coli* BL21(DE3) was used for expression and the protein was purified via IMAC from periplasmic preparations obtained via a standard osmotic shock procedure [31].

### Biosensor binding studies

Surfaces with immobilized CEA (Acro BioSystems, human CEA His_6_-tag, cat. hCE5-H5226, 2850 resonance units (RU)) produced in human embryonic kidney 293 (HEK293) cells and HSA (Sigma, 3400 RU) were prepared via standard amine coupling on a Biacore 3000 surface plasmon resonase (SPR) instrument (Cytiva) CM5 chip and used for the initial binding studies. Samples of His_6_-affibody-ABD fusions were injected at 200 nM for an initial characterization. Dilution series (1–2000 nM, 2-fold dilutions) of His_6_-C9-ABD and His_6_-C11-ABD were injected to estimate binding affinities. Monomeric C9-His_6_ and C11-His_6_ as well as head-to-tail dimers His_6_-C9-C9 and His_6_-C11-C11 were injected at 200 nM to investigate avidity effects from dimerization. For binding epitope analyses C9-C9-His_6_ was immobilized (800 RU) via amine coupling on a series S CM5 chip on a Biacore T200 instrument (Cytiva). Injection of CEA (50 nM; same as above) alone was compared with injections of CEA pre-mixed (1 h, RT) with 10-fold molar excess of C9-C9-His_6_, C11-C11-His_6_ or scFv MFE-23. The affinity of MFE-23 scFv was measured by injecting a dilution series (1–100 nM, 3-fold dilutions) over a freshly immobilized surface with CEA (2500 RU). Ala-mutants of affibody C9 (affibody-His_6_) were injected (200 nM) over 3000 RU of biotinylated CEA (Acro Biosystems, CE5-H82E0) captured on 2600 RU of streptavidin (Thermo Scientific, #21,122) immobilized via amine coupling on a Biacore T200 series S CM5 chip. Variants with no apparent binding were injected also at 2000 nM. Binding measurements to deglycosylated CEA were performed on a Biacore T200 using differently treated samples of biotinylated CEA (*ca.* 3000 RU) immobilized in separate flow cells of a CM5 chip with amine coupled streptavidin (*ca.* 2600 RU) followed by injection of affibody (C9-His_6_; 2.5–200 nM, 3-fold dilutions). Samples of untreated CEA, peptide-N-glycosidase F (PNGase F)- and heat-treated CEA and only heat-treated CEA were evaluated in parallel. PNGase-F (PNGase F glycan cleavage kit, Gibco, Cat. A39245) treatment was performed according to the manufacturer’s recommendations (1 h, 50 °C) prior to capture on streptavidin. For validation, a similar experiment was performed on a new chip using CEA that was deglycosylated at 37 °C overnight with a four-fold increased amount of PNGase-F (data not shown). All experiments were performed at 25 °C and 30 µl/min flow rate using phosphate-buffered saline (PBS) with 0,05 % Tween 20 (PBST), pH 7.4 as running buffer and 10 mM HCl for regeneration. Curve fitting was done using a Langmuir 1:1 binding model in the software BIAEval 3.2.1.

### Biotinylation

His_6_-C9-C9-Cys protein was biotinylated at the C-terminal cysteine using a maleimide-polethylene glycol2 (PEG2)-biotin conjugate (Thermo Fisher, Massachusetts, USA), according to the instructions from the supplier. After conjugation, the sample was purified using HPLC on a C18 column (Agilent, Santa Clara, USA) and analysed by matrix-assisted laser desorption/ionization time-of-flight MALDI TOF/TOF mass spectrometry (Sciex, Massachusetts, USA).

### Handling of clinical human pancreatic cancer samples

Human pancreatic cancer samples were collected from surgical resection specimens from patients who underwent surgery at the Karolinska University Hospital between March 2021 and March 2022. A piece of each tumor was sampled by a specialized pancreatic pathologist and thereafter divided in two parts. One part was fixed in formalin and embedded in paraffin, and the second part snap frozen in liquid nitrogen and stored at −80 °C until use. The series consisted of six pancreatic ductal adenocarcinomas (PDAC) and one pancreatic neuroendocrine tumor (PanNET). Clinicopathological characteristics are presented in Table 1.

**Table 1:** Clinicopathological data.

**ID-nr**	**Gender**	**Preoperative chemotherapy**	**Histological type**	**Grade of differentiation**	**Stage**
1	Female	No	PDAC	Poor	pT2 N1 M1
2	Male	No	PDAC	Poor	pT3 N2
3	Male	No	PDAC	Poor	pT2 N1 M1
4	Female	No	PDAC	Moderate	pT2 N2
5	Male	No	PanNET	Well	pT2 N1
6	Female	No	PDAC	Poor	pT2 N1
7	Male	No	PDAC	Moderate	pT3 N1

The study was approved by the Swedish Ethical Review Authority ("Etikprövnings-myndigheten", decision numbers 2012/1657–31/4, 2018–2654/32, 2019–00,788, and 2022–02,855–02). All patients were informed about the study before the intervention and provided their written consents. All study procedures were performed following the applicable guidelines and regulations.

### Affibody and immunohistochemical (IHC) staining

FFPE and frozen samples were cut into 5 µm and 4 µm thick sections, respectively. Stains were performed using an automated immunostainer (Leica BOND III or Ventana Benchmark Ultra), and in-house optimized protocols with anti-human CEA head-to-tail dimeric C9-C9-Bio affibody, mouse monoclonal CEA-II-7 (Dako, Agilent, Santa Clara, USA), mouse monoclonal CEA31 (Ventana, Roche Diagnostics, Indianapolis, USA) and rabbit polyclonal anti-CEA (Dako, Agilent). Information on the antibodies and staining protocols is summarized in Table 2. Specific protocols for the C9-C9-affibody used on both FFPE and frozen tissues are detailed in Table 3, while Table 4 outlines the protocols for monoclonal and polyclonal antibody reagents. After staining, the slides were digitalized using a Hamamatsu NanoZoomer scanner at 40X magnification. Two specialized pancreatic pathologists evaluated the digital slides and scored by consensus all the stainings in the following cell and tissue compartments: apical membrane, cytoplasm, and budding-like nests in the cancer cells, stromal cells, and normal epithelium. Scores were based on the percentage of stained cells and for diffuse scores (> 95 %) intensity was also included as a weight (moderate: 1.15, and strong: 1.30), multiplied by the extent of positive immunoreactivity. Data and statistical analyses were performed using R (v 4.2.1). The level of agreement between stains was calculated in a pairwise manner based on the intraclass correlation coefficient (ICC) using the irr package ("two-way" model, "agreement" type, "single" unit) and Bland-Altman plots depicting the bias and 95 % upper and lower limits of agreement. The ICCs were interpreted as poor: <0.5, good: 0.5–0.75, moderate: 0.75–0.9, and excellent: >0.90.

**Table 2:** Overview of the immunohistochemistry systems used in this study.

	**C9-C9-Bio**	**mAb II-7**	**mAb CEA31**	**Polyclonal Ab**
Source	This study	Dako (Agilent)	Ventana (Roche)	Dako (Agilent)
Product code	N/A	M7072	06433316001	A0115
Used concentration	3.2 µg/mL	0.518 µg/mL (diluted 1:400)	0.46 µg/mL (ready-to-use)	0.5 µg/mL (diluted 1:4000)
Platform	Bond-III Leica	Bond-III Leica	ultraBenchmark Ventana	ultraBenchmark Ventana
Detection	Bond Intense R	DAB Refine	DAB ultraView	DAB ultraView

**Table 3:** Details of staining protocols used for the C9-C9-Biotin reagent.

	**Affibody reagent Frozen sections**	**Affibody reagent FFPE sections**
1	Bond wash [0.3 min]	Dewax (deparaffinization) [0.3 min]
2	1 % BSA-PBS [15 min]	HIER (pretreatment) with EDTA pH 9 [20 min]
3	Peroxide block [5 min]	Bond wash [0,3 min]
4	Streptavidin block [15 min]	1 % BSA-PBS [15 min]
5	Biotin block [15 min]	Peroxide block [5 min]
6	Marker (His_6_-C9-C9-Cys-PEG-Biotin) [45 min]	Streptavidin block [15 min]
7	Streptavidin-HRP (Bond Intense R) [8 min]	Biotin block [15 min]
8	Mixed DAB [5 min]	Marker (His_6_-C9-C9-Cys-PEG-Biotin) [45 min]
9	Hematoxylin [5 min]	Streptavidin-HRP (Bond Intense R) [8 min]
10	**–**	Mixed DAB [5 min]
11	**–**	Hematoxylin [5 min]
**Material for biotin-based staining (affibody reagent):** Bond Intense R Detection: DS9263 Diluent: Bond primary diluent: AR9352 Biotin solution: 30011 Vector Laboratories Streptavidin solution: 30088 Vector Laboratories

**Table 4:** Details of staining protocols used for the monoclonal and polyclonal antibody reagents.

	**Antibody reagents Frozen sections, 0,2 µm**		**Antibody reagents FFPE sections, 0,1 µm**	
	**DAB Refine (II-7)**	**DAB ultraView (CEA31, pAb)**	**DAB Refine (II-7)**	**DAB ultraView (CEA31, pAb)**
1	Marker (II-7) [30 min]	Marker (CEA31 or pAb) [30 min]	Dewax (deparaffinization) [0.3 min]	Dewax (deparaffinization) [0.3 min]
2	Post Primary [15 min]	Peroxide block [5 min]	HIER (pretreatment) with EDTA pH 9 [20 min]	HIER (pretreatment) with EDTA pH 9 [20 min]
3	Peroxide block [5 min]	Polymer) [15 min]	Marker (II-7) [30 min]	Marker (CEA31 or pAb) [30 min]
4	Polymer [15 min]	Mixed DAB [10 min]	Post Primary [15 min]	Peroxide block [5 min]
5	Mixed DAB [10 min]	Hematoxylin [10 min]	Peroxide block [5 min]	Polymer [15 min]
6	Hematoxylin [10 min]		Polymer [15 min]	Mixed DAB [10 min]
7	**–**		Mixed DAB [10 min]	Hematoxylin [10 min]
8	**–**		Hematoxylin [10 min]	

### Molecular modelling

Molecular models of complexes between CEA and affibodies were generated by AlphaFold3 (https://golgi.sandbox.google.com/) using the Uniprot entry P06731 (CEACAM5) and the amino acid sequences of affibody clones C9 and B6 as input. The generated models were analysed in PyMOL (Schrödinger, Germany).

### Mammalian cell lines

All cell lines were purchased from American Type Culture Collection ATCC (Manassas, VA, USA). The BxPC3 (pancreatic cancer) was maintained in an RPMI 1640 growth medium supplemented with 20 % fetal bovine serum (FBS) (Sigma-Aldrich, St. Louis, MO, USA). The HT-29 and the LS174T cells (colon cancer) were maintained in an RPMI 1640 growth medium supplemented with 10 % FBS. The growth medium for all cell lines was supplemented with antibiotics (100 international units (IU) penicillin and 100 μg/mL streptomycin), Biochrom GmbH, Berlin, Germany). The cells were cultured in a humidified incubator at 37 °C in a 5 % CO_2_ atmosphere.

### Radiochemistry

^99m^Tc was obtained as pertechnetate [TcO4]^−^ by elution of an Ultra-TechneKow ^99m^Tc/ ^99^Mo generator (Mallinckrodt) with sterile 0.9 % sodium chloride (Mallinckrodt, Petten, The Netherlands). A Cyclone Storage Phosphor System (CR-35 BIO Plus, Elysia-Raytest, Bietigheim-Bissingen, Germany) was used for analysis of radiochemical yield and purity using instant thin layer chromatography (iTLC). Kits enabling facile and reproducible labelling were prepared and lyophilized, each containing 1 mg Sn(II) chloride dihydrate, 5 mg gluconic acid sodium salt and 100 μg EDTA [2]. For labelling, the content of one kit was dissolved in 120 μL degassed PBS and added to 30–40 μg of an affibody fusion protein solution, followed by an eluate containing 100–200 MBq of ^99m^Tc[TcO_4_]^−^. The preparation was vortexed and incubated at 37 °C for 75 min. Thereafter, the radiochemical yield was measured. When necessary, the ^99m^Tc-labelled conjugates were purified using the size-exclusion NAP-5 columns, pre-equilibrated and eluded with PBS. A cysteine challenge test was performed to assess the stability of the label, involving incubation of the radiolabelled fusion protein at 37 °C for 15 min in a 300-fold molar excess of the l-cysteine before determining the fraction protein-bound activity by instant thin layer chromatography (ITLC) [22]

### *In vitro* evaluation of ^99m^Tc-labelled proteins

Specific binding was assessed on cell lines having a high, medium or low CEA expression; BxPC3, LS174T and HT-29, respectively. Briefly, LS174T (10^6^ cells/well), BxPC3 (7 × 10^5^ cells/well), and HT-29 (7 × 10^5^ cells/well) were seeded in 6-well plates and incubated for 24 h at 37 °C until they attached. One set of control dishes was incubated with 500 nM of non-labelled proteins for 30 min, to saturate/block CEA, while the test set was incubated with an equal volume of fresh media with or without HSA. Thereafter, the radiolabelled proteins were added to a final concentration of 5 nM to both sets of dishes. After 1 hour of incubation at 37 °C, the media was aspirated; the cells were washed with 2 ml of media, detached using trypsin, collected and counted using an automated cell counter. The cell-associated activity was measured using a gamma-counter with a NaI (Tl) detector (2480 Wizard, Wallac, Finland). Each measurement was repeated in triplicates.

### Biodistribution and tumour targeting

All animal experiments were performed in accordance with national legislation on laboratory animal protection, and the study was approved by the local Ethics Committee for Animal Research. Four mice per data point were used in the biodistribution experiments. An intraperitoneal injection of Ketalar/Rompun was used to euthanize the mice. BALB/C nu/nu mice were subcutaneously implanted on the hind legs with approximately 8 × 10^6^ BxPC3 or 5 × 10^6^ HT-29 cells. Experiments were performed two-three weeks after implantation, at a tumour size of approximately 150 mm^3^. The average animal weight was 19 ± 1 g at the time of the experiment. Biodistribution samples were taken from mice bearing BxPC3 xenografts sacrificed four hours after injection. Each mouse was intravenously injected in the tail vein with 6 µg (70 kiloBequerel (kBq)) [^99m^Tc]Tc-C9-C9-EYEC, in 100 μL PBS containing 1 % BSA. The injected protein mass was adjusted using non-labelled C9-C9-EYEC. The animals were euthanized four hours post injection. Blood, salivary glands, lung, liver, stomach without content, spleen, pancreas, large intestine, kidneys, tumour, muscle, bone, and the remaining carcass were collected, weighed and the amount of radioactivity measured using a gamma-counter (2480 Wizard, Wallac). CEA-dependent tumour uptake *in vivo* was visualized four hours after injection. The injected amount was 6 µg (9 MBq). To verify saturable uptake of [^99m^Tc]Tc-C9-C9-EYEC in the BxPC3 xenograft, one mouse was subcutaneously pre-injected with 1 mg unlabelled C9-C9-EYEC at one hour before intravenous injection of [^99m^Tc]Tc-C9-C9-EYEC. After euthanization the mice were immediately placed in the nanoScan single-photon emission computed tomography (SPECT/CT) scanner (MedisoMedical Imaging Systems, Budapest, Hungary), as described earlier [33].

## Results

### Identification of two families of CEA-specific affibodies

**Fig. 1:**
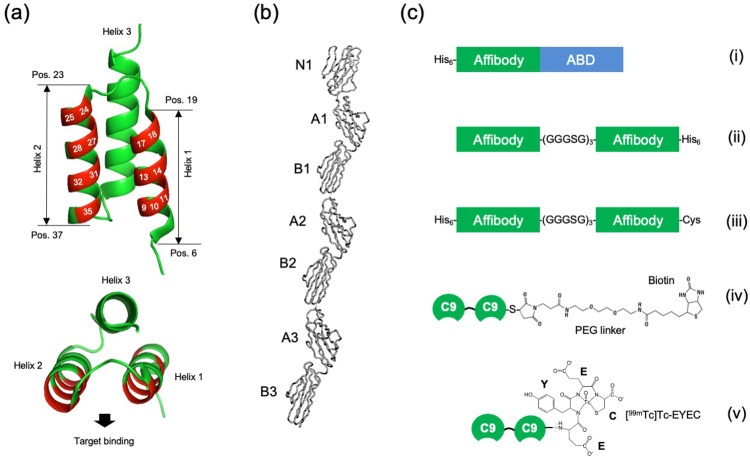
**Schematic description of the main proteins included in this study. (a)** Overall three-dimensional structure of the 58 aa affibody scaffold, showing the three-helix bundle organization. The 14 positions in helices 1 and 2 that are subjected to combinatorial randomization to construct libraries are highlighted in red and numbered. **(b)** Molecular model of the seven-domain CEA 3D-structure 1E07.pdb previously validated using solution scattering data [7]. **(c)** Schemes of the different affibody formats used within the present study including (i) His_6_-affibody-ABD, where ABD is a 46 aa serum albumin binding domain, (ii) affibody-affibody-His_6_, (iii) His_6_-affibody-affibody-Cys, (iv) a dimeric anti-CEA affibody C9 construct conjugated to maleimide-PEG-biotin (C9-C9-Bio) and (v) a dimeric anti-CEA affibody C9 construct containing an EYEC extension allowing for site-specific labeling with [^99m^Tc]Tc.

Two combinatorial phagemid-based affibody phage libraries (Lib-1 and Lib-2) were used for *in vitro* selection campaigns against a recombinant biotinylated CEA target protein containing all seven Ig-like domains (corresponding to aa 35–685) (Fig. 1**a and b**). The two libraries differed regarding the variant of albumin-binding domain (ABD)-phage coat protein 3 construct used as gene fusion partner in the phagemids, and the helper phages used for their preparation (**Fig. S1**). During selections, streptavidin bead-captured phage-target complexes were eluted in parallel using either low pH (pH 2.6) or trypsin cleavage, to potentially broaden the diversity of the output in terms of characteristics of affibody-target interactions. After four cycles of selections, a total of 94 output clones were randomly picked from all tracks and analysed for CEA, HSA, BSA and streptavidin binding using a microtiter-scale monoclonal phage-ELISA assay. Phage-ELISA analyses showed that a majority of the 192 analysed clones displayed clear binding to CEA and the positive control HSA, with very low background binding to the negative controls (**Fig. S2**). A total of 96 top-ranked phage-ELISA positive clones from both libraries, and from both acid and trypsin elution tracks, were sequenced

The sequences of the affibody gene inserts of all 96 clones allowed for identification of several sequence clusters by phylogenetic analyses (**Fig. S3**). Some clones had been highly enriched during selections and were represented several times, including clones B6 (14x), D2 (24x) and D4 (9x) from Lib-1, as well as C9 (17x) and F7 (8x) from Lib-2, whereas other clones were represented by single hits. Some clones were found in outputs from both acid and trypsin elution tracks. Interestingly, 13 clones contained tryptophan residues at both positions 9 and 10 in helix 1, and four additional clones had a phenylalanine and a tryptophane residue at positions 9 and 10, respectively. Furthermore, two clones (B6 and H11) comprised either WW or FW combinations at positions 27 and 28 in helix 2 (**Fig. S4)**. All 19 unique variants were reformatted as His_6_-affibody-ABD fusion constructs (Fig. 1**c–i**), produced as soluble proteins in *E. coli*, and their binding to CEA analysed using SPR biosensor technology. Separate injections of the His_6_-affibody-ABD fusions at a 200 nM concentration over a sensor chip surface containing immobilized CEA revealed a wide distribution of response signal strengths and binding kinetics (**Fig. S5a**). Binding responses of the fusion proteins to an HSA-containing sensor chip surface, via the included ABD moiety, indicated that the injected concentrations were similar and that none of the fusion proteins formed multimers or aggregates that would have altered the 1:1 binding pattern (**Fig. S5b**). Two of the variants, C9 and C11, displayed the highest binding responses and slowest off-rate kinetics, and were therefore selected for further characterization. Monovalent 1:1 binding affinity (dissociation constant, K_D_) of these variants to CEA was determined from injections of serial dilutions to *ca*. 200 nM, albeit with somewhat different binding profiles, where the C9 variant showed slower off-rate kinetics (**Fig. S6**).

**Fig. 2:**
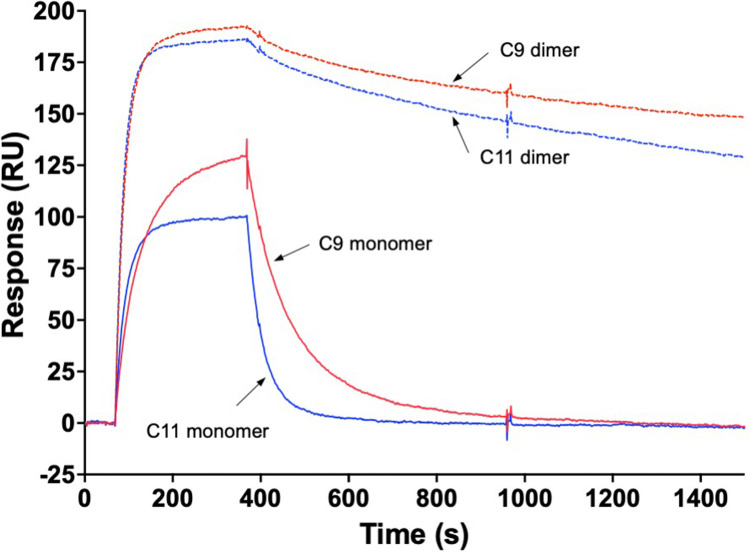
**Homodimeric affibody constructs bind with significantly higher apparent affinity to CEA compared to monomers.** Proteins were injected at 200 nM concentrations over a sensor chip surface containing immobilized human CEA protein. **(a)** Binding of monomeric C9-His_6_ and C11-His_6_ proteins, and **(b)** binding of homodimeric His_6_-C9-C9-ABD and His_6_-C11-C11-ABD proteins to CEA. Note the significantly slower off-rate kinetics for homodimeric affibody constructs, via avidity effects.

To investigate if the apparent affinity to CEA could be increased via avidity effects, head-to-tail dimer fusions were constructed (Fig. 1**c–ii**) and evaluated for their binding affinities to sensor chip-immobilized CEA. The results demonstrated that both dimer constructs C9-C9-His_6_ and C11-C11-His_6_, in which the two affibody moieties were connected via a 15 aa flexible (GGGSG)_3_ linker, displayed significantly higher overall binding responses. Furthermore, both these head-to-tail dimer constructs displayed markedly slower off-rate kinetics compared to their monomeric counterparts, indicating a clear avidity effect and thus that both affibody moieties were functional when produced in this dimeric format (Fig. 2).

### All 19 clones bind in the N-terminal portion of CEA

**Fig. 3:**
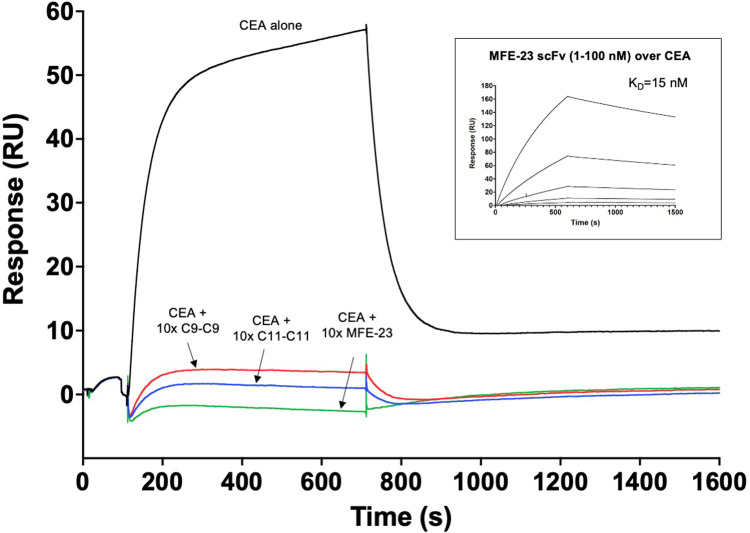
**Biosensor binding competition experiments reveal the high specificity of dimeric affibody constructs to CEA.** Binding of either free CEA or CEA mixed with a 10-fold molar excess of either C9-C9-His_6_, C9-C9-His_6_ or anti-CEA MFE-23 scFv proteins to a sensor chip surface containing immobilized C9-C9-His_6_, demonstrated that all three analytes blocked binding of CEA to C9-C9-His_6_. **Inset:** Analysis of the binding of a concentration series (100–1 nM, 3-fold dilutions) of free anti-CEA MFE-23 scFv to a sensor chip surface containing immobilized CEA.

Using a sensor chip surface containing immobilized C9-C9-His_6_ protein, a series of epitope binning experiments were performed. First, injections of either free CEA or CEA pre-mixed with a 10-fold molar excess of either C11-C11-His_6_ or C9-C9-His_6_ proteins over the sensor chip surface, revealed that pre-mixing of CEA with C11-C11-His_6_ abolished the binding signal seen for free CEA to immobilized C9-C9-His_6_, indicating the presence of overlapping epitopes on CEA for C9 and C11 (Fig. 3). As expected, a similar blocking was seen after pre-mixing CEA with C9-C9-His_6_ (self-blocking control). These findings prompted us to investigate also the other 17 variants (as ABD fusions) in the same manner, and all showed a similar capability to block the binding between CEA and C9 (data not shown).

Further, the binding profile obtained from injection of free CEA over C9-C9-His_6_ resembled the 1:1 binding profile obtained in the reversed format using a monovalent C9 fusion protein (Fig. 2). This indicates that CEA, although composed by multiple domains with high sequence homology **(Fig. S7)**, contains only a single epitope for the C9 and C11 affibodies, as otherwise slower off-rate kinetics from di- or multi-site binding would have been seen.

A previously described anti-CEA scFv denoted MFE-23 was included in further binding competition experiments. This scFv has been previously proposed to bind to a less glycosylated part of the surface of CEA, localised within the first two N-terminal domains N1 and A1, supported by experimental data [7,38]. In the present study, the functionality of an in-house produced MFE-23 was first confirmed in a direct binding experiment to immobilized CEA, where it was found to bind with an affinity of around 15 nM (Fig. 3, **inset**). Pre-mixing CEA with a 10-fold molar excess of MFE-23 scFv abolished binding of CEA to C9-C9-His_6_ (Fig. 3).

Taken together, this series of experiments indicate that all 19 affibody clones bind to an epitope within the two N-terminal domains of CEA that overlaps with the binding site of the MFE-23 scFv.

### Alanine scanning studies of clone C9 reveals the importance of individual residues for affinity to CEA

**Fig. 4:**
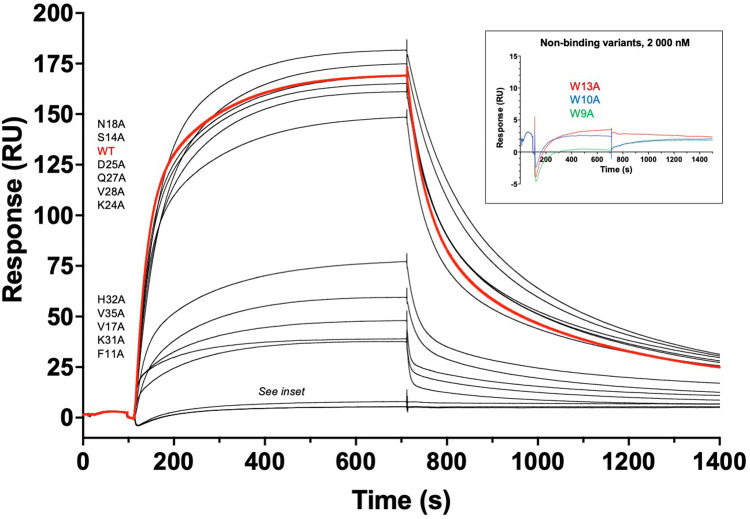
**Substitution of specific tryptophan residues in C9 abrogates binding to CEA.** The binding capacity of 14 alanine substituted C9 variants was compared to parental C9 (C9 wild type (WT), bold red trace). The binding of each monovalent affibody-His_6_ protein to immobilized CEA is presented. The same concentration of 200 nM was used for all variants. Variants N18A, S14A, D25A, Q27A, V28A and K24A showed essentially retained binding affinity, whereas variants H32A, V35A, V17A, K31A and F11A displayed markedly reduced binding responses. The absence of detectable binding at 200 nM for the three variants W9A, W10A and W13A was confirmed by a second analysis using 2000 nM (inset).

To investigate the relative importance of individual amino acid residues on the affibody binding surface for recognition of CEA, a set of 14 individual alanine mutated variants corresponding to the positions randomized during the library construction were cloned, expressed, purified as C9*_Ala mutant_*-His_6_ constructs (Fig. 1**c–iii and S8**), and subjected to CEA binding studies with unmodified C9 affibody as a reference. The results demonstrated that the mutations S14A, N18A, K24A, D25A, Q27A and V28A did not significantly affect binding, while F11A, V17A, K31A, H32A and V35A resulted in slightly decreased binding affinities. In contrast, an alanine substitution at any of the residues W9, W10 or W13 abrogated binding to CEA (Fig. 4). Circular dichroism spectroscopy analyses of these alanine-substituted variants did not indicate any substantial differences in their secondary structure contents compared to the wild type C9 affibody (**Fig. S9**). Further, thermal melting point analyses were performed on the affibody variants in which each of the three tryptophan residues W9, W10 and W13 and residues F11 and K31 were mutated to alanine. While the W9A and W10A substitutions reduced the thermal stability by 5–8 °C, a substitution at position 13 to alanine resulted in a decreased thermal stability of close to 20 °C (**Fig. S10**). Interestingly, alanine substitutions at F11A or K31A resulted in slightly increased thermal stability. Taken together, these results indicate that the almost complete loss of CEA binding observed for two of the three tryptophan mutants (W9A and W10A) does not correlate with a corresponding loss of secondary structure, and that instead effects from alterations in their binding interfaces to CEA most likely caused the dramatic loss of binding. However, for the third tryptophan mutant (W13A) it cannot be excluded that the reduced thermal stability affected its binding functionality, although the structural integrity is very high also for this variant during the binding analysis when performed at 25 °C.

### Clone C9 binds with high affinity to a non-glycosylated epitope on CEA

The importance of the three tryptophan residues for recognition of CEA by C9 prompted us to investigate the influence of glycosylation for these interactions, since tryptophans have been previously reported to be heavily overrepresented in proteins that recognize carbohydrate structures [24]. To this end, streptavidin-coated sensor chip surfaces were employed for binding studies between the head-to-tail dimeric C9-C9-His_6_ affibody and biotinylated CEA that was either untreated or treated with PNGase F, an enzyme that effectively removes N-linked oligosaccharides from proteins. Our results demonstrated that binding of C9 to CEA was not significantly affected by the removal of all carbohydrates, indicating that the C9/CEA interaction does not involve carbohydrates (**Fig. S11**). Parallel experiments demonstrated that binding of MFE-23 scFv to CEA, shown to bind competitively with C9, was also unaffected by PNGase F-mediated deglycosylation (**Fig. S12**).

**Fig. 5:**
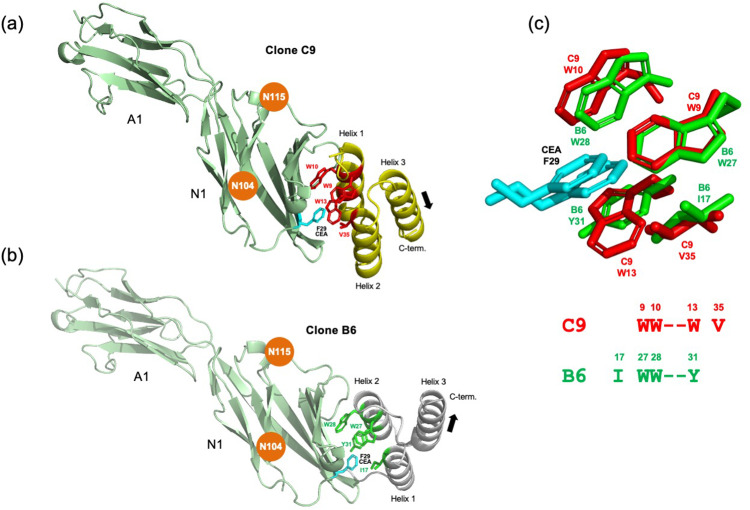
**AlphaFold3 models of affibody-CEA complexes.** AlphaFold3-advanced [1,27] was used to model complexes between CEA and either of two anti-CEA affibodies. ***(a)****Affibody C9:CEA complex.* Showing one of several generated models suggesting that a region at the very end of domain N1 contained the epitope for the C9 affibody. Centrally positioned in the binding interface is a phenylalanine 29 (F29) from CEA, whose side chain appears to interact with all three aromatic side chains of the W9-W10-W13 cluster in helix 1, and V35 in helix 2 of the C9 affibody. The proposed epitope is also free from postulated N-glycosylations, of which the closest are predicted to reside on positions N104 and N115 (highlighted in brown) at a certain distance from the epitope. ***(b)****Affibody B6:CEA complex.* The clone B6 was also analysed in the same manner. This clone also contains an aromatic cluster, W27-W28-Y32, but located in helix 2 which, together with I17 located in helix 1 also appears to be involved in binding to the side chain of F29 in CEA. Notably, the affibody is orientated in the opposite direction to align the cluster of clone B6 located on helix 2 which is anti-parallel to helix 1. ***(c****) Alignment of aromatic clusters of C9 and B6.* An alignment of the models of the aromatic clusters in C9 and B6, together with the F29 of CEA, shows a near perfect pairwise overlap of sidechains involved. Also shown is the alignment of the aliphatic residues V35 (C9) and I17 (B6), both appearing to contribute to the hydrophobic binding interface.

To investigate possible binding sites in CEA, AlphaFold3 [1,27] was used to produce molecular models of complexes formed between the N-terminal domains N1-A1 of CEA and two of the anti-CEA affibodies (C9 and B6). The highest ranking proposed molecular model for theC9:CEA complex suggests that a region at the very end of domain N1 contains the epitope for the C9 affibody (Fig. 5**a**). The model indicates that the side chain of the phenylalanine residue F29, positioned centrally within the proposed CEA binding interface, forms interactions with the aromatic side chains of the three tryptophans W9, W10 and W13 in affibody C9, as well as with the valine residue V35 positioned in helix 3, resulting in significant van der Waals, hydrophobic and π-π interactions. The proposed molecular model thus provides a structural explanation for the lack of binding observed upon mutating any of these three tryptophans to an alanine. The proposed epitope is also free from N-glycosylation, distant from the closest sugar predicted to reside on positions N104 and N115 (highlighted in brown). The N-terminal N1 domain does not share any significant homology with other parts of the sequence, which is well in line with our results indicating a single binding site in CEA **(Fig. S7)**. Taken together, the proposed molecular model is in agreement with all our experimental data, in which i) a binding site within the N-terminal domain of CEA that ii) is present only in a single copy in the full-length CEA protein, iii) does not involve carbohydrates and iv) depends on the co-localization of three tryptophans and additional hydrophobic amino acids.

### The second affibody family contains the same binding motif

Affibody B6 contains a similar aromatic cluster as compared to C9 constituted by W27-W28-Y32, which is instead located on helix 2 (Fig. 5**b**). An AlphaFold3 model of the B6:CEA complex indicates that this cluster, together with the isoleucine residue I17 in helix 1 (overlapping to V35 in clone C9) appears to form hydrophobic, van der Waals and π-π interactions with the side chain of the CEA residue F29. However, the tryptophane/tyrosine cluster in B6 is located on helix 2, which is positioned anti-parallelly to helix 1. Therefore the affibody in the B6:CEA complex points in the opposite direction, and is rotated approximately 180° compared with the C9:CEA complex. A structural superposition of the C9:CEA and B6:CEA complex models (Fig. 5**c**) reveals a near perfect (root mean square deviation (RMSD)=0,45 Å) pairwise overlap of the aromatic clusters in clones C9 and B6. The interaction formed by these two clusters with the side chain of residue F29 in CEA suggests a strong selection pressure for such clustered arrangements whether these appear on helix 1 or helix 2 of the affibody domain.

### Unambiguous detection of CEA in human pancreatic cancer samples using dimeric C9-C9 affibody

The high affinity binding to CEA observed for the dimeric C9-C9 construct (Fig. 2) prompted us to investigate its potential use for the detection of CEA in complex biological samples. To this end, a homodimeric His_6_-C9-C9-Cys construct (Fig. 1**c-iii**) was assembled to assess the immunohistochemical capacity of this construct to detect CEA in human pancreatic cancer specimens. The C-terminal cysteine residue of the construct was recruited for site-specific biotinylation using maleimide-PEG-biotin (Fig. 1**c–iv and S13**) so as to enable detection via a streptavidin-HRP conjugate. Our SPR analyses demonstrated that the His_6_-C9-C9-Cys-PEG-Biotin conjugate (C9-C9-Bio) bound to CEA with an affinity of approximately 2 nM (**Fig. S14**), well in line with our initial binding experiment results using the unconjugated head-to-tail dimeric C9-C9-His_6_ protein.

**Fig. 6:**
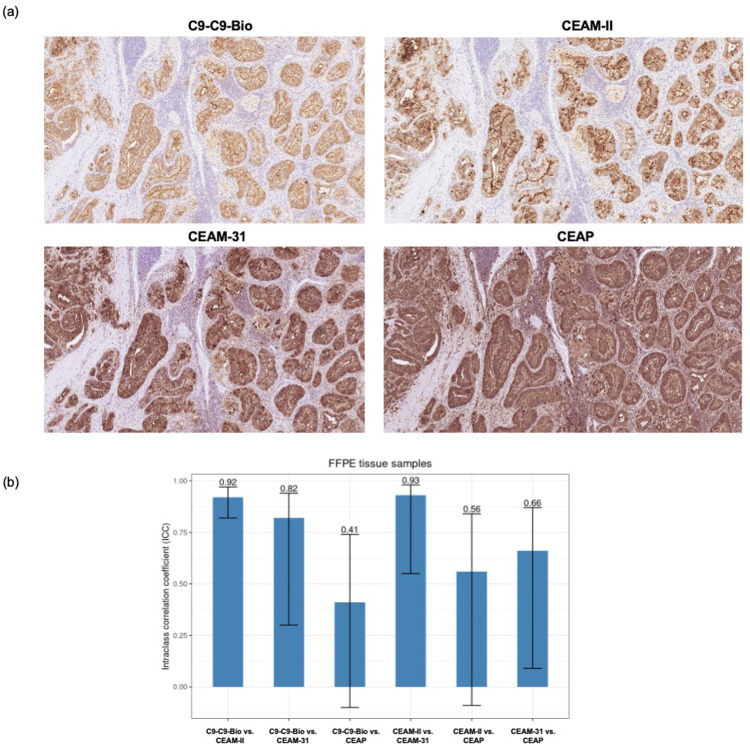
**The homodimeric C9-C9 affibody allows for unambiguous detection of CEA in FFPE samples. (a)** Representative photomicrograph of CEA detection with the dimeric C9-C9-Bio reagent and the clinical-grade monoclonal and polyclonal antibodies. **(b)** Intraclass correlation coefficients (ICC) as measurement of agreement in CEA detection scoring between the dimeric C9-C9-Bio reagent and the monoclonal and polyclonal antibodies.

**Fig. 7:**
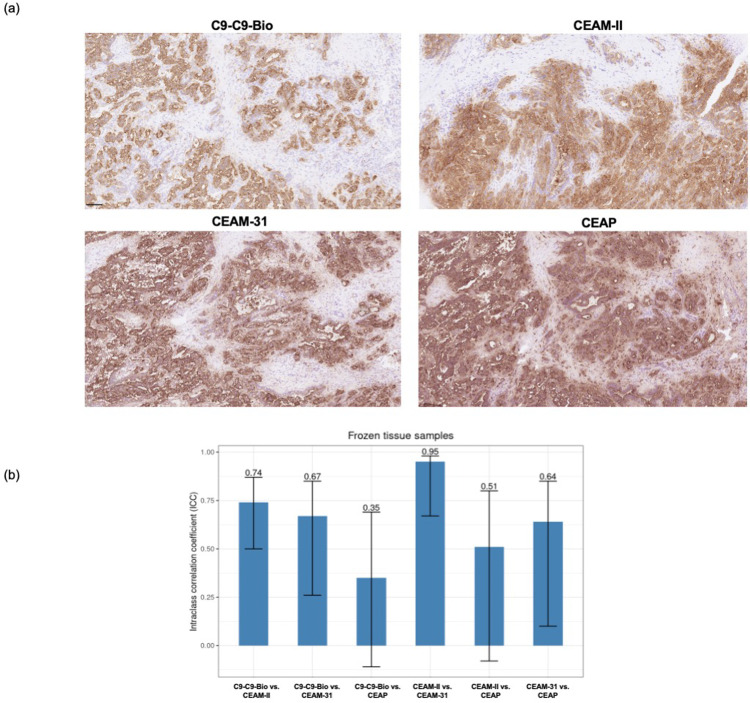
**CEA detection in frozen samples using the homodimeric C9-C9 affibody. (a)** Representative photomicrograph of CEA detection with the dimeric C9-C9-Bio reagent and the clinical-grade monoclonal and polyclonal antibodies. **(b)** Intraclass correlation coefficients (ICC) as measurement of agreement in CEA detection scoring between the dimeric C9-C9-Bio reagent and the monoclonal and polyclonal antibodies.

Depending on the affinity reagent used and the sample type (frozen or formalin-fixed paraffin-embedded, FFPE), different instrumentation and protocols were applied (Table 2, Table 3, Table 4). The performance of the C9-C9-Bio conjugate for adequate detection of CEA within tissue samples was compared to both monoclonal anti-CEA antibodies II-7 from Dako/Agilent, and CEA-31 from Ventana/Roche (Table 2, Table 4), as well as with a polyclonal reagent (Dako/Agilent). It should be noted that all comparators are used routinely in clinical diagnostics. Two pancreas pathologists assessed independently the different stains first at a qualitative level. The overall staining pattern was highly consistent between C9-C9-Bio and the two monoclonal antibodies in regard to the extent and the spatial location of the stained cancer cells, as well as negative reactivity in non-malignant cells (stromal, inflammatory, and normal epithelium), and the absence of background staining (Figs. 6**A** and 7**A**). However, the C9-C9-Bio reagent produced a highly accurate and defined CEA detection with an apical membrane accentuation, superior to the monoclonal antibodies. The staining pattern of C9-C9-Bio was very similar to that of CEA-II, while CEA-31 yielded a more saturated stain with cytoplasmic reactivity, more in line with the results usually obtained with the polyclonal antibody reagent. The polyclonal product produced a poorly specific staining with reactivity in stromal cells and background.

The stains were further evaluated quantitatively in a pairwise manner based on the pathologists scores. C9-C9-Bio displayed an excellent and moderate intraclass correlation coefficient (ICC) agreement on FFPE tissues with the monoclonal antibodies CEA-II and CEA-31, respectively. Accordingly, its agreement was poor with the staining obtained with the polyclonal reagent, showing a diffuse staining (Fig. 6**B**). These results were highly consistent with the qualitative assessment. The agreement of ICCs between C9-C9-Bio and the monoclonal antibodies were lower but in the good range for frozen tissues (Fig. 7**B**). Similarly, Bland-Altman analysis demonstrated that the agreement in CEA detection was highest between C9-C9-Bio and monoclonal CEA-II, followed by monoclonal CEA-31 (bias = −7 and -19, respectively), and that the performance of C9-C9-Bio was superior in FFPE compared to frozen tissues.

### Binding of ^99m^Tc-labelled C9-C9 to living human cancer cells is target-specific

Site-specific labelling with ^99m^Tc was performed on a C9-C9 derivative produced with the short Tc- chelating peptide EYEC (Fig. 1**c-v)**. The labelled protein was purified by size-exclusion chromatography to obtain a radiochemical purity exceeding 95 %. The stability of the label was confirmed by a cysteine challenge test (data not shown). The binding specificity of the ^99m^Tc-labelled constructs to CEA-expressing cancer cells was confirmed using BxPC-3 and LS174T cells, with HT29 as a negative control. The cell-bound activity was significantly higher with the CEA-positive cell lines BxPC3 and LS174T (3018 ± 85 and 3291 ± 1153 CPM/10^5^ cells, *n* = 3) compared with the CEA-negative HT29 cell line (142 ± 19 CPM/10^5^ cells, *n* = 3). Saturation of the target on CEA-positive BxPC3 and LS174T cells by adding a large excess of unlabeled proteins brought down the activity bound to CEA-expressing cancer cells to background levels (144 ± 77 and 710 ± 160 CPM/10^5^ cells, *n* = 3), whereas the value for the CEA-negative HT29 remained at background level (129 ± 19) **(Fig. S15)**.

### Biodistribution and tumor targeting

The biodistribution of radioactivity was determined four hours after injection in BALB/C nu/nu mice xenografted with BxPC3 pancreatic cancer cells, which natively have high CEA-expression. (Table 5) A low activity remained in circulation (blood) at this time point. The concentration of the radioactivity in the tumor was higher than in any non-excretory organ. The highest concentration was found in the kidneys, and the second highest in the liver, although lower. In addition, some radioactivity was noticed in the contents of the gastrointestinal tract. The percentage of activity was low, showing that only a small fraction of activity was excreted via bile.

**Table 5:** **Distribution of [^99m^Tc]Tc-C9-C9-EYEC in mice bearing BxPC3 xenografts 4h after injection**. Data for the rest of the intestines are presented as the uptake in the whole sample including content.

	**Uptake,%ID/g**	**Tumor-to-organ ratio**
**Blood**	0.33±0.03*	9.6 ± 1.3*
**Salivary gland**	1.19±0.07*	2.6 ± 0.1*
**Lung**	0.62±0.04*	5.1 ± 0.7*
**Liver**	1.3 ± 0.1*	2.4 ± 0.1
**Spleen**	0.7 ± 0.1	4.4 ± 0.5*
**Stomach**	1.2 ± 0.1*	2.8 ± 0.4*
**Pancreas**	0.30±0.04*	10.6 ± 1.0*
**Large intestine**	1.0 ± 0.2*	3.1 ± 0.5
**Kidney**	112±7*	0.028±0.004*
**Tumor**	3.1 ± 0.2*	
**Muscle**	0.17±0.02*	18.9 ± 0.7*
**Bone**	0.31±0.01	10.1 ± 0.4*
**Rest of intestines**	5.5 ± 0.6	

**Fig. 8:**
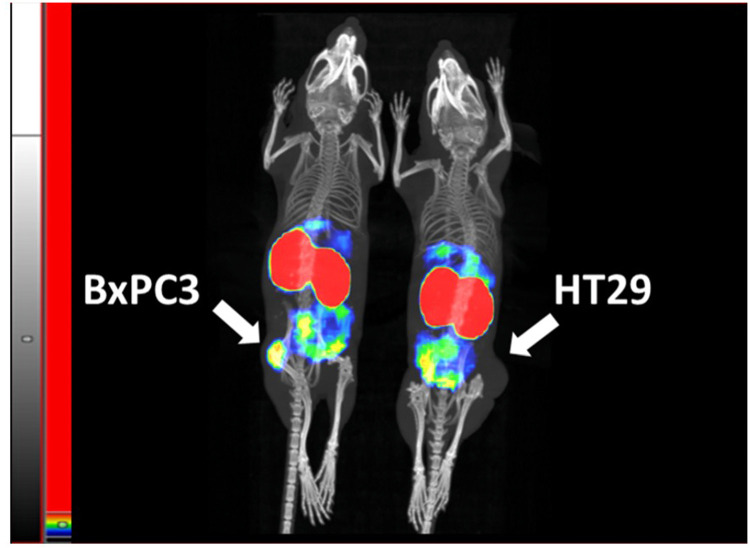
Visualization of CEA-dependent uptake of [^99m^Tc]Tc-C9-C9 in human cancer xenografts in mice (color). The animals were injected with the same activity of [^99m^Tc]Tc-C9-C9 and the image of both animals was acquired 4 h after injection. Maximum Intensity Projection (MIP) is presented. The threshold of the linear color intensity scale is adjusted to the first red pixel in BxPC3 xenograft with high CEA expression.

**Fig. 9:**
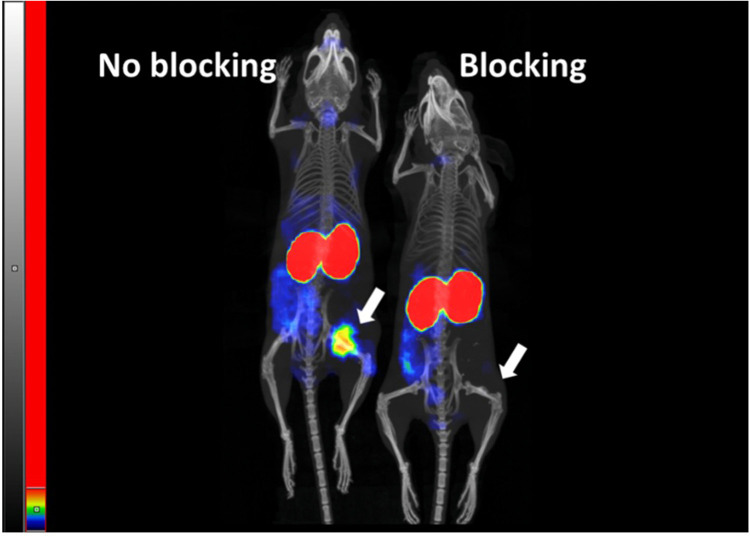
Visualization of saturable uptake of [^99m^Tc]Tc-C9-C9 in human cancer BxPC3 xenografts in mice. The animals were injected with the same activity of [^99m^Tc]Tc-C9-C9 and the image of both animals was acquired 4 h after injection. Pre-injection of a large excess of non-labelled C9-C9-EYEC was used in the blocking experiment. Maximum Intensity Projection (MIP) is presented. The threshold of the linear color intensity scale is adjusted to the first red pixel in BxPC3 xenograft in the animal with non-blocked tumor.

Imaging was performed both in mice xenografted with high CEA-expressing BxPC3 cells (above), or HT29 cells not expressing CEA. The BxPC3 tumors are clearly visualized, yielding a higher signal than all non-excretory tissues, whereas HT29 tumors do not appear over background levels from normal non-excretory tissues (Fig. 8). A blocking experiment was performed providing further support for highly selective CEA targeting *in vivo*, demonstrating a large reduction in the tumor signal BxPC3 xenografts when injection of [^99m^Tc]Tc-C9-C9 was done one hour after injection of a 100-fold excess of non-labeled C9-C9 (Fig. 9). The high accumulation in the kidneys stands out in these experiments. MicroSPECT/CT imaging demonstrated that the activity accumulates in the renal cortex **(Fig. S16)**.

## Discussion

In the study, nineteen unique CEA binders were identified by phage display, grouping in two families by sequence comparison. Characterization of the strongest binding affibody C9 by an alanine scan showed that two, and possibly three, tryptophans in the first of the two anti-parallel helices of the C9 affibody were critical for binding. As tryptophans have been previously described as overrepresented in carbohydrate-binding proteins [24], this raised the question whether the C9 epitope comprised glycans. However, retained binding to PNGase F-treated CEA suggested that no major contributions from protein-carbohydrate interactions are involved. This finding was structurally rationalized by creating and analyzing an AlphaFold 3 molecular model of the complex formed between CEA and the affibody C9, suggesting a binding site that involves the side chain of F29 in CEA, protruding from the non-glycosylated tip of the outmost domain N1. The side chain of residue F29 is surrounded by a cluster of three tryptophan residues in C9, with other hydrophobic residues also contributing to the formed interface. A highly similar aromatic-rich motif is also found in the second family of affibody binders, but are instead here located on the second helix. A molecular model of a representative clone (B6) from this sequence family in complex with CEA suggests a high specificity for the same epitope, and that even if the affibodies C9 and B6 are oriented in opposite directions, their hydrophobic clusters overlap strictly, with a overall RMSD of 0.45 Å. Thus, all 19 unique affibody molecules display a similar aromatic-rich binding motif, indicating a strong preference for binding to the epitope localized on the exposed tip of domain N1 in CEA.

A dimeric head-to-tail affibody construct C9-C9 was created to enhance the apparent affinity of the binding interaction when assessing clinically relevant applicability. The dimeric affibody construct was provided with a unique C-terminal cysteine used for coupling with maleimide biotin, making C9-C9-Bio suitable for evaluation as an immunohistochemical reagent. The dimeric affibody construct produced a highly accurate and defined CEA detection with an apical membrane accentuation, which was similar to that of monoclonal CEA-II and superior to that of a polyclonal CEA antibody in terms of specificity and absence of background staining. In contrast CEA-31 showed a more saturated staining pattern with cytoplasmic reactivity. The successful results from stains of FFPE samples were unexpected given the extensive processing involving formalin fixation, paraffin embedding, dewaxing, heat-induced epitope retrieval (HIER) treatment and peroxide blocking. To the best of our knowledge, this is the first study in which a non-immunglobulin affinity protein has been used to provide a highly accurate CEA staining in human pancreatic cancer FFPE samples.

Previous studies investigating the use of monoclonal antibodies for CEA detection have reported diverging results [14,29,51], with some antibodies yielding high sensitivity and specificity, while others display low sensitivity due to cross-reactivity with normal tissues. The current study's results using the C9-C9-Bio reagent are consistent with those that reported high sensitivity, specificity, and absence of background staining with monoclonal antibodies, such as CEA-II [15,34]. It is plausible that the small size of the C9-C9-Bio reagent of *ca*. 16.5 kDa, compared to antibodies with a size of 150 kDa, contributed to a more precise and antigen-localized enzymatic staining pattern. In addition, the obtained resolution of the CEA location is influenced by the overall size and composition of all secondary/tertiary reagents recruited to form the detection complex. In this respect, the principles for HRP-based detection used for the C9-C9-Bio and mAbs detection differ significantly. The C9-C9-Bio detection is based on a single secondary reagent, SA-HRP, whereas the CEA-bound antibodies are detected by using up to two layers of heavily conjugated secondary antibody reagents (denoted post primary and polymer antibodies) to amplify the signal, resulting in a final deposition of HRP reporter enzymes in a much larger complex, which altogether contributes to a lower resolution of the actual location of the CEA antigen. In short, the fine definition of the CEA localization demonstrates that the C9-C9-Bio construct could be a valuable tool for pathologists to assess a cell type and cellular compartment-specific staining pattern, and rule out unspecific staining, which would contribute to improved accuracy in cancer diagnosis. Furthermore, the recombinant nature of the C9 affibody construct opens up for the design of a second generation of reagents and alternatives to the biotin/SA-HRP detection used in this study, including *e.g*. multimeric constructs with more than two affibody units, and direct genetic fusion to HRP as has been previously described for other recombinant affinity domains [41].

The addition of the EYEC amino acid sequence at the C-terminus of C9-C9 formed a peptide-based chelator, permitting a stable clinically validated labelling with ^99m^Tc [10]. [^99m^Tc]Tc-C9-C9 demonstrated saturable CEA expression-dependent binding to human cancer cell lines *in vitro*, showing that the binding specificity was preserved after labelling. *In vivo* experiments demonstrated a rapid localization in tumor and clearance from the rest of the body, except for excretory organs. The high values in kidney four hours post injection (112±7 %ID/g, 33 ± 2 %ID/organ) demonstrated that this protein follows the typical excretion pattern of other small proteins, *i.e.* filtration via glomureli and reabsorption in proximal tubuli of the renal cortex [16]. The cortical localization of activity in the kidneys was confirmed by the microSPECT/CT imaging. The total activity in the liver and intestines including content (7.2 ± 0.4 % of the ID) was more than four-fold lower than in the kidneys, showing that hepatobiliary excretion plays a minor role in clearance of [^99m^Tc]Tc-C9-C9.

The imaging experiments provided a clear visualization of the BxPC3 xenografts four hours post-injection and demonstrated that the uptake in CEA-positive BxPC3 xenografts was significantly higher than in xenografts of the CEA-negative HT-29. The target-specific accumulation in BxPC3 xenografts was further underscored by the demonstration of the saturable character of [^99m^Tc]Tc-C9-C9 uptake. The concentration of radioactivity in the tumor was 10-fold higher than in normal pancreas. Typical sites for metastases of pancreatic cancer are the liver, lymph node, and lung [35]. Colorectal cancer, which also expresses CEA, metastasizes into the liver and lungs [52]. Also in these organs, the concentration of radioactivity was several-fold lower than in tumors, suggesting that imaging of CEA-expressing metastases would be possible in a clinical setting.

The favorable *in vivo* targeting properties of [^99m^Tc]Tc-C9-C9 are in agreement with previous experiences with other affibody constructs, tested for *in vivo* radionuclide molecular diagnostics and used *e.g.* for clinical imaging of HER2-expressing tumors [13,46] ^99m^Tc]Tc-C9-C9 compares favorably with immunoglobulin-based imaging agents that bind specifically to CEA, including scFv-Fc H310A showing 2.8 ± 1.1 %ID/g 20 h post injection, and a tumor-to-blood ratio of 5.7 [20]. A CEA-imaging probe, which is based on another promising immunoglobulin-based scaffold, single domain antibody (sdAb, nanobody), has also been reported [48]. Although nanobodies have a molecular weight similar to the weight of C9-C9, the CEA-binding [^99m^Tc]Tc-nanobody displayed a tumor-to-blood ratio of less than two, and a high hepatic uptake. Thus, it was clearly inferior to [^99m^Tc]Tc-C9-C9.

Another report on CEA-binding affibodies was published while this manuscript was drafted [40]. The sequences of the presented affibodies show no similarity to ours, and do not comprise the hydrophobic motif that was identified in the cohort of affibodies identified in the present study, indicating that these other affibodies may bind to a separate site on CEA. These affibodies also stained formalin-fixed tissue sections *in vitro* and were rapidly renally cleared *in vivo*, but due to the difference in imaging modality it is impossible to directly compare the tumor *versus* normal organ uptake values with the current study. Furthermore, we investigated in our case the *in vitro* and *in vivo* properties of a dimeric affibody construct, making comparisons with this other study even more difficult.

In summary, we have developed a family of affibody molecules that bind with high specificity and affinity to the outer domain of CEA, showing favorable properties. Furthermore, our data suggest that different derivatives of the CEA-binding C9 affibody may be developed for various clinical applications, including IHC and medical imaging, but also as a targeting agent for directing various therapeutic modalities towards CEA-expressing tumors.

## CRediT authorship contribution statement

**Johan Nilvebrant:** Writing – review & editing, Writing – original draft, Visualization, Validation, Supervision, Software, Resources, Methodology, Investigation, Formal analysis, Data curation, Conceptualization. **Carlos Fernández Moro:** Writing – review & editing, Writing – original draft, Visualization, Validation, Supervision, Resources, Project administration, Methodology, Investigation, Formal analysis, Data curation, Conceptualization. **Eleftherios Papalanis:** Visualization, Validation, Methodology, Investigation, Formal analysis. **Masih Ostad Novin:** Investigation. **Haozhong Ding:** Investigation. **Ruonan Li:** Investigation. **Maryam Oroujeni:** Investigation. **Arun Selvam:** Investigation. **Béla Bozóky:** Investigation. **Torbjörn Gräslund:** Supervision. **Timea Szekerczes:** Investigation. **Tatiana Sandalova:** Formal analysis. **Hugh Salter:** Writing – review & editing, Writing – original draft, Investigation, Conceptualization. **Adnane Achour:** Writing – review & editing, Writing – original draft, Visualization, Validation, Supervision, Resources, Project administration, Methodology, Investigation, Funding acquisition, Formal analysis, Data curation, Conceptualization. **Vladimir Tolmachev:** Writing – review & editing, Writing – original draft, Visualization, Validation, Supervision, Software, Resources, Project administration, Methodology, Investigation, Funding acquisition, Formal analysis, Data curation, Conceptualization. **Mikael Björnstedt:** Writing – review & editing, Writing – original draft, Visualization, Validation, Supervision, Resources, Project administration, Methodology, Investigation, Funding acquisition, Formal analysis, Data curation, Conceptualization. **Per-Åke Nygren:** Writing – review & editing, Writing – original draft, Visualization, Validation, Supervision, Resources, Project administration, Methodology, Investigation, Funding acquisition, Formal analysis, Data curation, Conceptualization.

## Declaration of competing interest

The authors declare the following financial interests/personal relationships which may be considered as potential competing interests: The new anti-CEA affinity proteins described in the manuscript are subject of a patent application, involving the following inventors: JN, CFM, HS, AA, MB and PÅN.

## Appendix Supplementary materials

Image, application 1
