# Supplementary material for: An anti-CEA affibody showing high-definition staining in human pancreatic cancer tissue sections and selective tumor targeting in vivo
Source: Transl Oncol. 2025 Aug 28;61:102512. doi: 10.1016/j.tranon.2025.102512 (PMC12410180; doi:10.1016/j.tranon.2025.102512)
Supplement: Supplementary file 1 [file mmc1.docx]

Supplementary information

**An anti-CEA affibody showing high-definition staining in human pancreatic cancer tissue sections and selective tumor targeting *in vivo***

Johan Nilvebrant^1#^, Carlos Fernández Moro^2,3#^, Elefterios Papalanis^4^, Masih Ostad Novin^2^, Haozhong Ding^1^, Ruonan Li^1^, Maryam Oroujeni^4^, Arun Selvam^3^, Béla Bozóky^2^, Torbjörn Gräslund^1^, Timea Szekerczes^3^, Tatiana Sandalova^5,6^, Hugh Salter^3^, Adnane Achour^5,6^, Vladimir Tolmachev^4^, Mikael Björnstedt^2,3*^ and Per-Åke Nygren^1,7*^

1) Dept of Protein Science, AlbaNova University Center, KTH Royal Institute of Technology, SE-144 21 Stockholm, Sweden

2) Department of Clinical Pathology and Cancer Diagnostics, Karolinska University Hospital F46, SE-141 86, Stockholm, Sweden

3) Division of Pathology, Dept of Laboratory Medicine, Karolinska Institute, SE-141 52 Huddinge, Sweden

4) Department of Immunology, Genetics and Pathology, Uppsala UniversitySE-751 85, Uppsala, Sweden

5) Science for Life Laboratory, Department of Medicine, Karolinska Institute, SE-171 65 Solna, Sweden

6) Division of Infectious Diseases, Karolinska University Hospital, SE-14186 Stockholm, Sweden

7) Science for Life Laboratories, Karolinska Institute, SE-171 65 Solna, Sweden

#) Shared first authors

*) Co-corresponding authors

**Figure S1. Schematic description of the phagemid and helper phage used in Lib-2.**

**(a)** Block diagrams of elements in the phagemid library member expression cassette and the protein 3 encoded by the KM13 helper phage, respectively. The phagemid expression cassette contains several elements including i) a signal peptide from the *E. coli* OmpA protein, (ii) an affibody library member, (iii) the albumin binding domain (ABD), (iv) the trypsin-sensitive sequence, GARRAG, (v) a suppressible amber (TAG) stop codon, and (vi) the full-length M13 phage protein 3. The KM13 helper phage encodes the engineered full-length protein 3, into which the trypsin-sensitive sequence GRGA has been introduced between domains D2 and D3 of the protein. **(b)** During phage particle assembly in the *E. coli* periplasm, protein 3 molecules encoded from both the phagemid (including the affibody and ABD elements) and the helper phage, compete with each other. The system is intentionally designed for a preferential incorporation of protein 3 from the helper phage, resulting in a large fraction of non-affibody-displaying phage particles. However, in the fraction of phages that actually display affibody-ABD-protein 3 fusion proteins, the display is monovalent (one fusion protein per phage particle), facilitating affinity selections between affibodies and target proteins on a 1:1 interaction basis, avoiding avidity effects from cooperative binding events that could otherwise contribute to biases in the selection pressure. The introduction of trypsin-sensitive sites in both the phagemid and the helper phage facilitates both trypsin-mediated elution of target-binding phage particles, typically from target-containing beads, and a simultaneous elimination of unwanted non-affibody displaying phage particles during selections. Whereas affibody-ABD fusion displaying phage particles after trypsin cleavage still carries a full-length protein 3 protein that can mediate *E. coli* infection, necessary for the preparation of enriched phages for next selection cycle, non-affibody-ABD fusion displaying phage particles will only display a truncated protein 3 (only domain 3) after trypsin cleavage, that is unable to mediate infection. It should also be noted that during phage display selections, the combined use of phagemids and helper phage results in the production of four types of phage particles, of which only two are presented here.


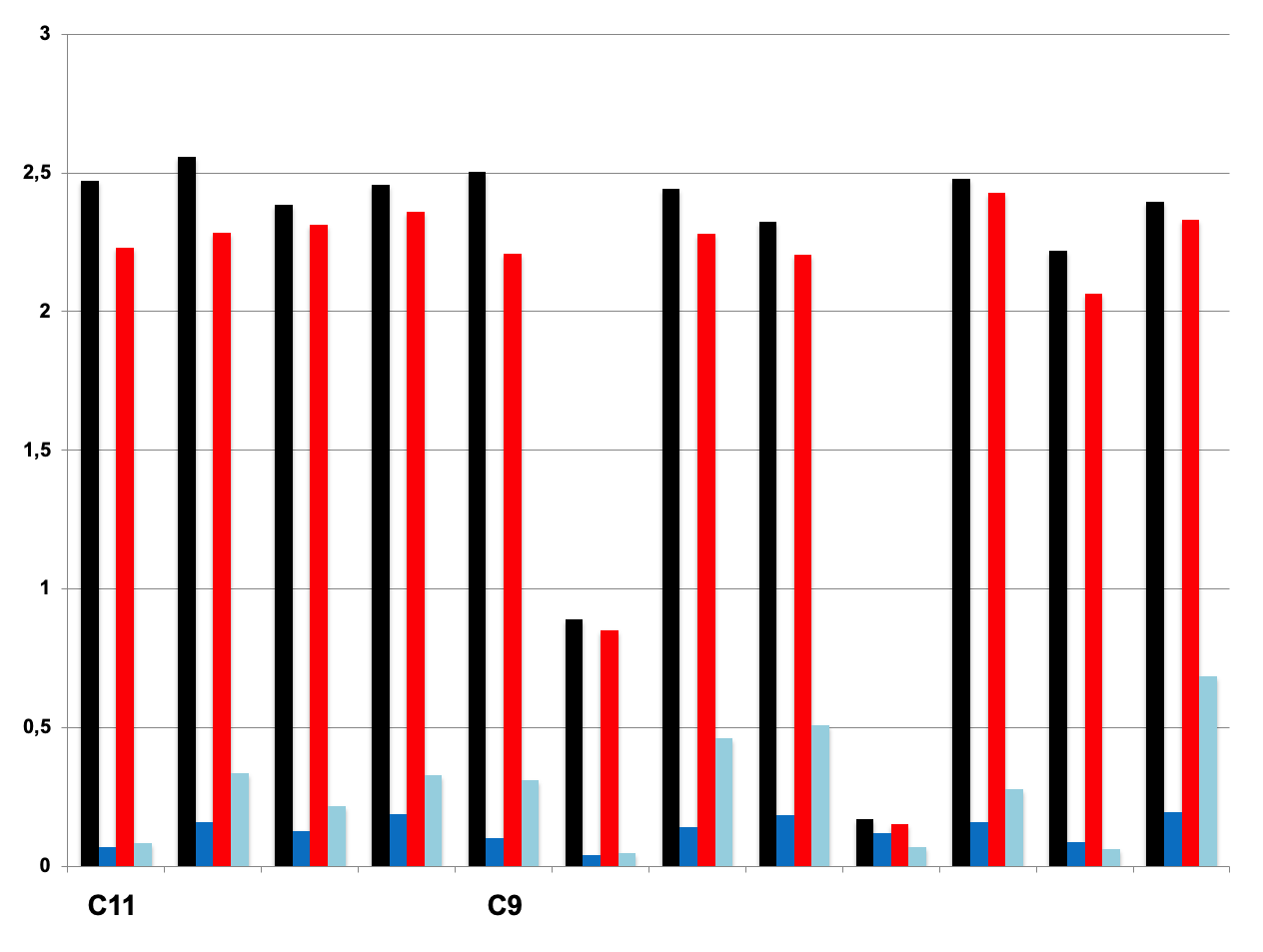


**Figure S2. Initial analyses of target specificity using monoclonal phage ELISA.**

Representative data from phage-ELISA analyses of individual phage stock preparations of clones after the fourth round of selection. Phage particles were assessed for their binding capacity to four ligands: (i) HSA (positive control, black bars), (ii) streptavidin (negative control, dark blue bars), (iii) CEA (target, red bars) and (iv) BSA (negative control, light blue bars). Clones C11 and C9 are highlighted.


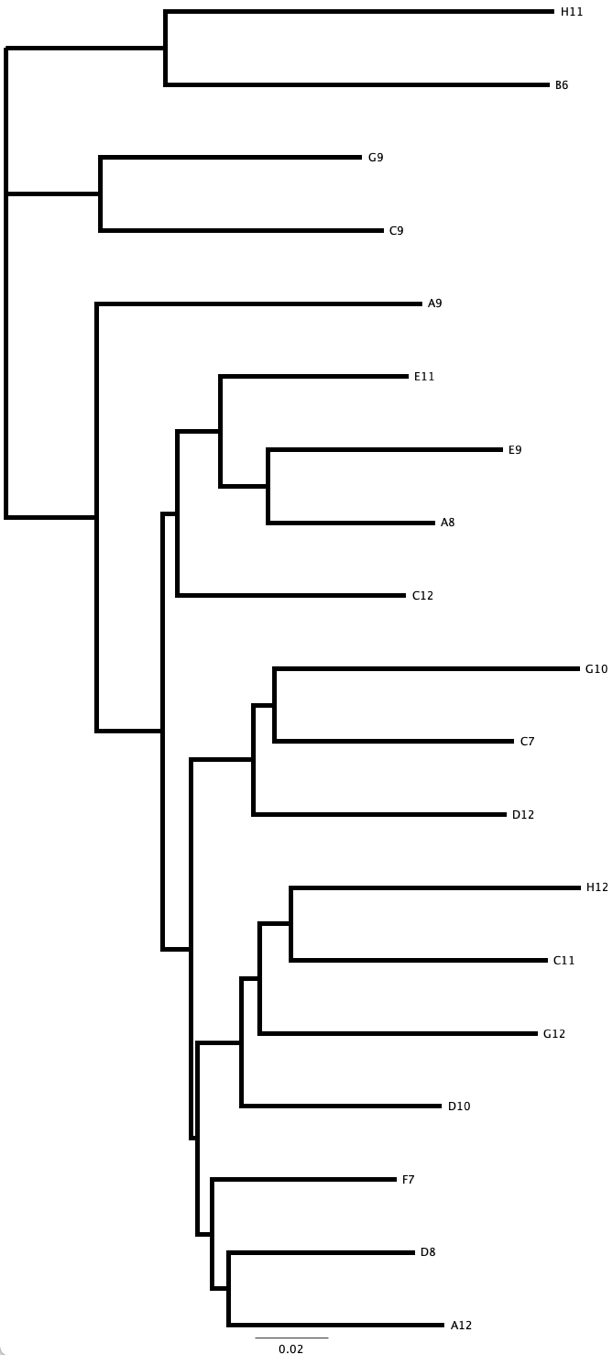


**Figure S3.** **Phylogenetic analysis of the 19 unique phage-ELISA positive clones.**

**
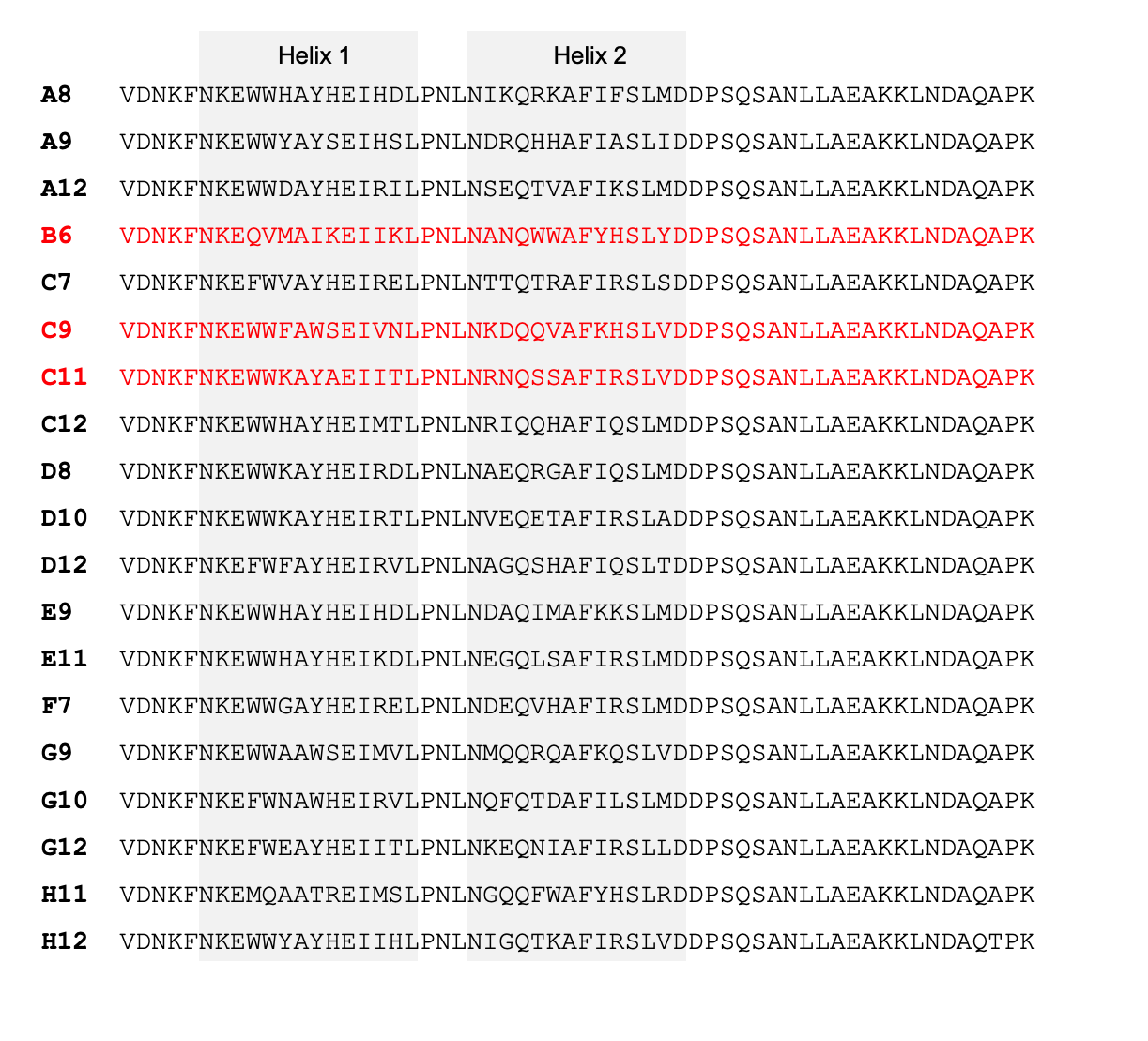
**

**Figure S4.** **Amino acid sequences of the 19 identified unique affibody clones.**

An alignment of 19 unique clones reveals the clustering of aromatic residues within either the first or the second randomized helix (shaded regions). The sequences of clones C9, C11 and B6 discussed within the main text are highlighted in red.

**
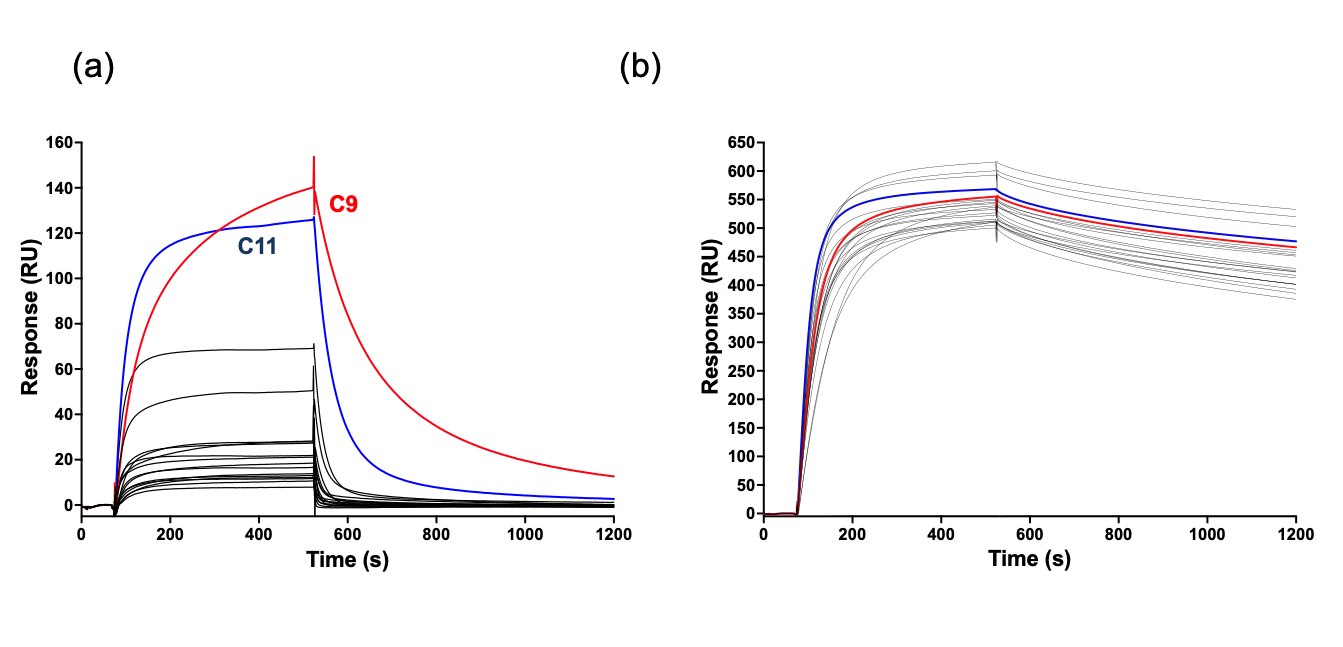
**

**Figure S5.** **Biosensor binding analyses of the 19 unique anti-CEA affibody clones.**

Individual His_6_-affibody-ABD fusion proteins corresponding to the 19 unique clones were injected at a concentration of 200 nM over sensor chip surfaces containing either hCEA **(a)** or HSA **(b)**. The results from the CEA sensor chip surface demonstrate that all 19 clones bind to hCEA, but with different characteristics. Clones C9 (in red) and C11 (in blue) displayed the strongest binding responses, and the slowest off-rate kinetics. The results from injections of the same 19 samples over the HSA sensor chip surface confirmed that all variants were injected at approximately similar concentrations, and that none of these variants formed aggregates.

*
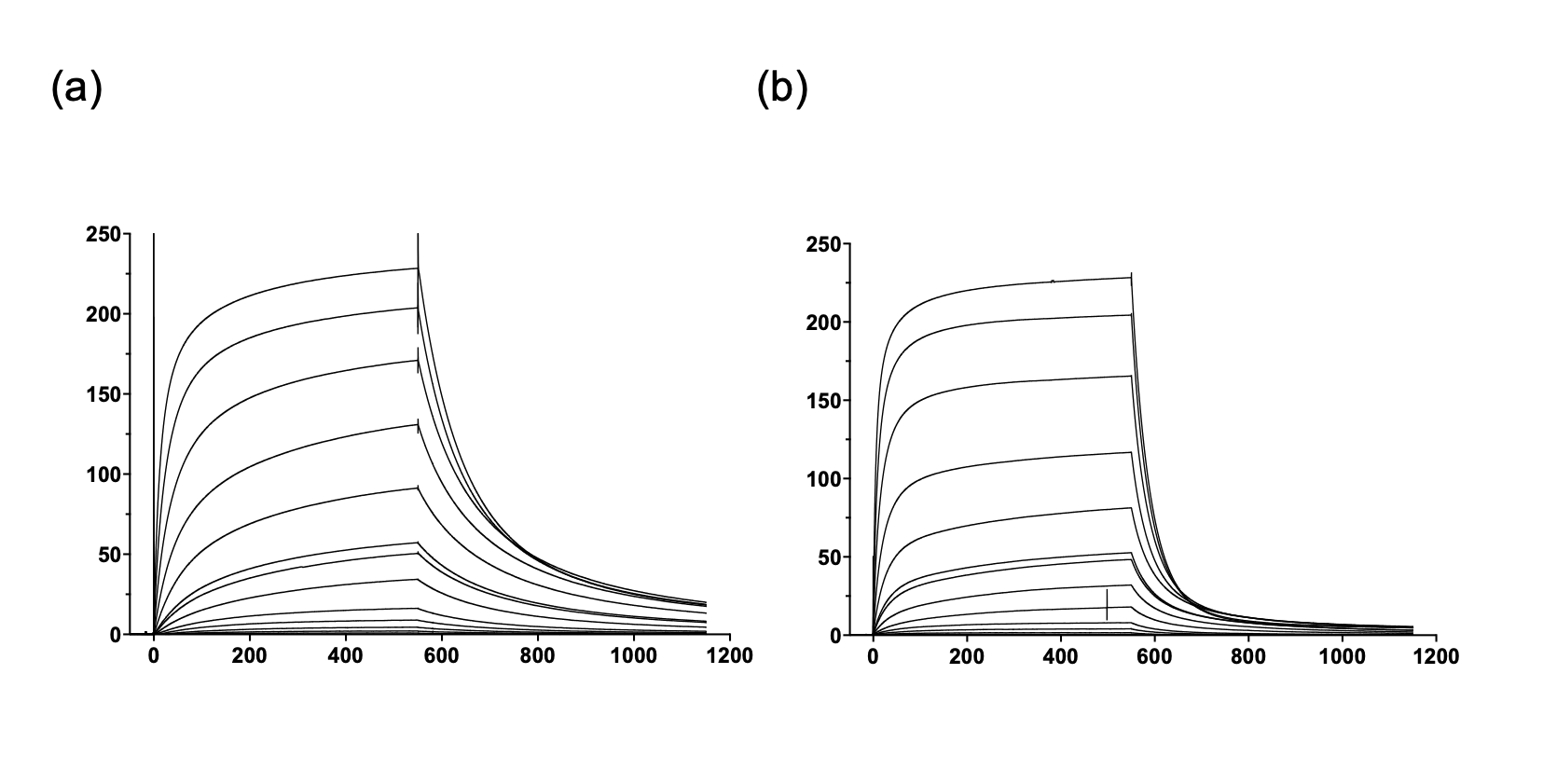
*

**Figure S6.** **SPR analysis of C9 C11 binding kinetics.**

A series of concentrations of C9-ABD (a) or C11-ABD (b) fusion proteins ranging between 1-2000 nM were injected over a sensor chip surface containing immobilized CEA. Using BIAevaluation software for data fitting, the affinities (K_D_) for both fusion proteins could be determined to ca. 200 nM.

**Figure S7. Analysis of repeat sequences in CEA (CEACAM5).**

The analysis was performed using the Dotmatcher software (www.bioinformatics.nl/cgi-bin/emboss/dotmatcher) and shows how the A and B domains (residues 145-675), but the not N-terminal N1 domain (residues 35-144), of CEA share regions of high homology.


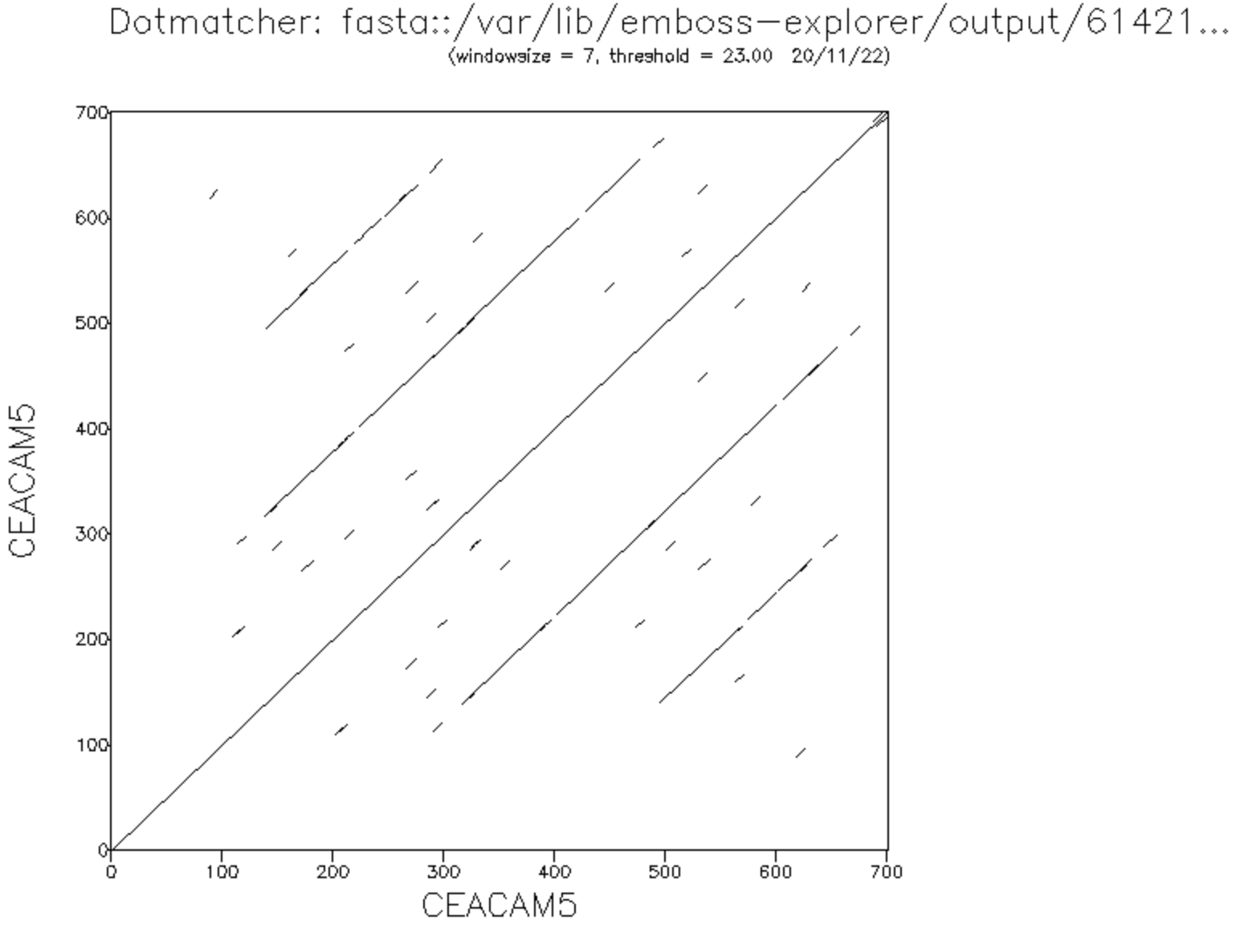


**
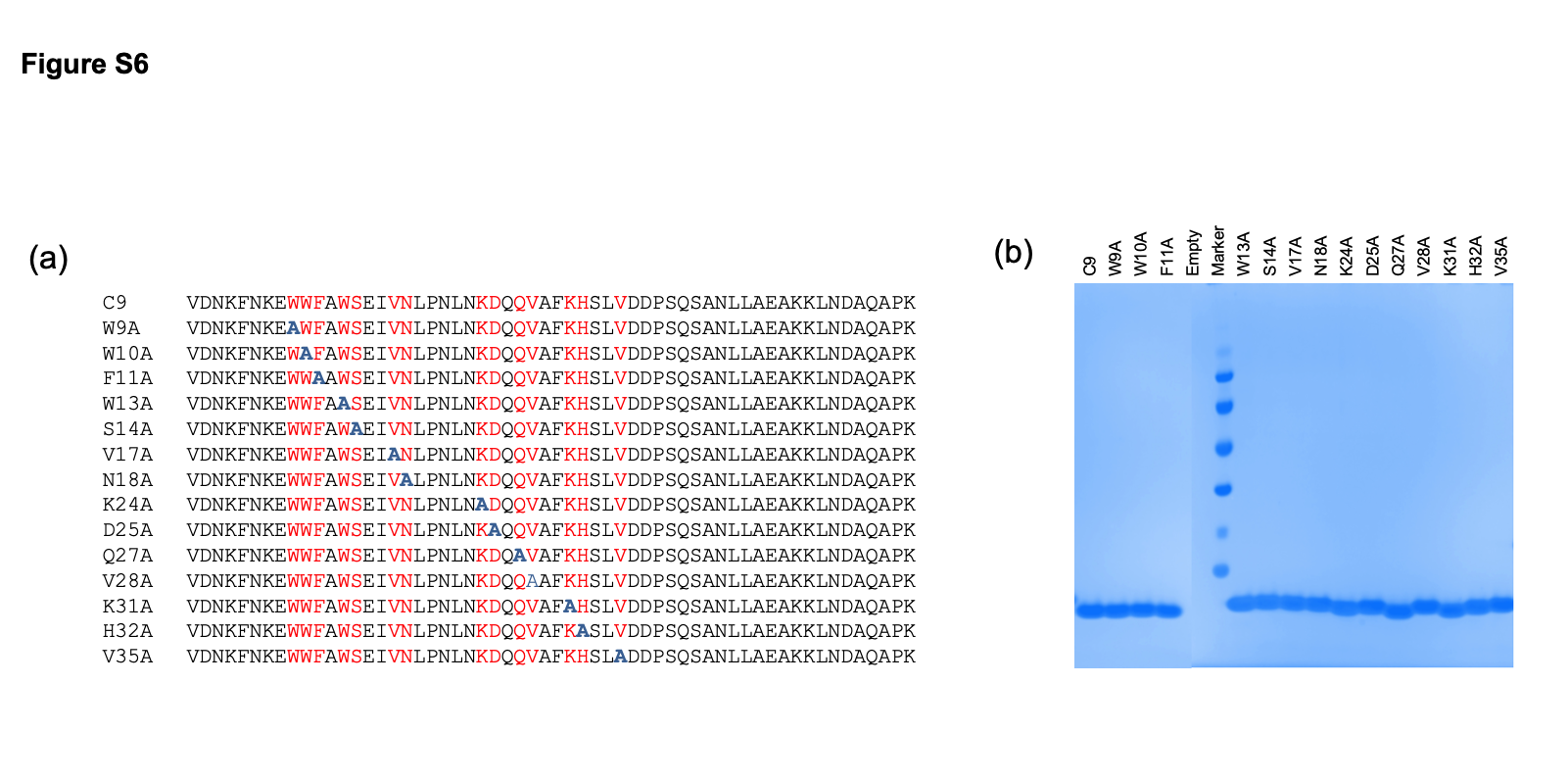
**

**Figure S8.** **Design and production of 14 individual alanine mutated variants of the affibody clone C9.**

**(a)** The amino acid sequences of individual alanine mutated variants of clone C9 are listed, with the parental C9 sequence included as a reference (top row). Each of the 14 affibody positions (9, 10, 11, 13, 14, 17, 18, 24, 25, 27, 28, 31, 32 and 35, all highlighted in red), were mutated to alanine and included in the combinatorial randomization to construct the libraries. **(b)** Wild type C9 clone and the 14 alanine mutants were produced as affibody-His_6_ fusion proteins in *E. coli* and purified from cell lysates under native conditions via IMAC, The purity of each produced construct was assessed in chromatograms (data not shown) and sodium dodecyl sulfate–polyacrylamide gel electrophoresis (SDS-PAGE) analysis.

**
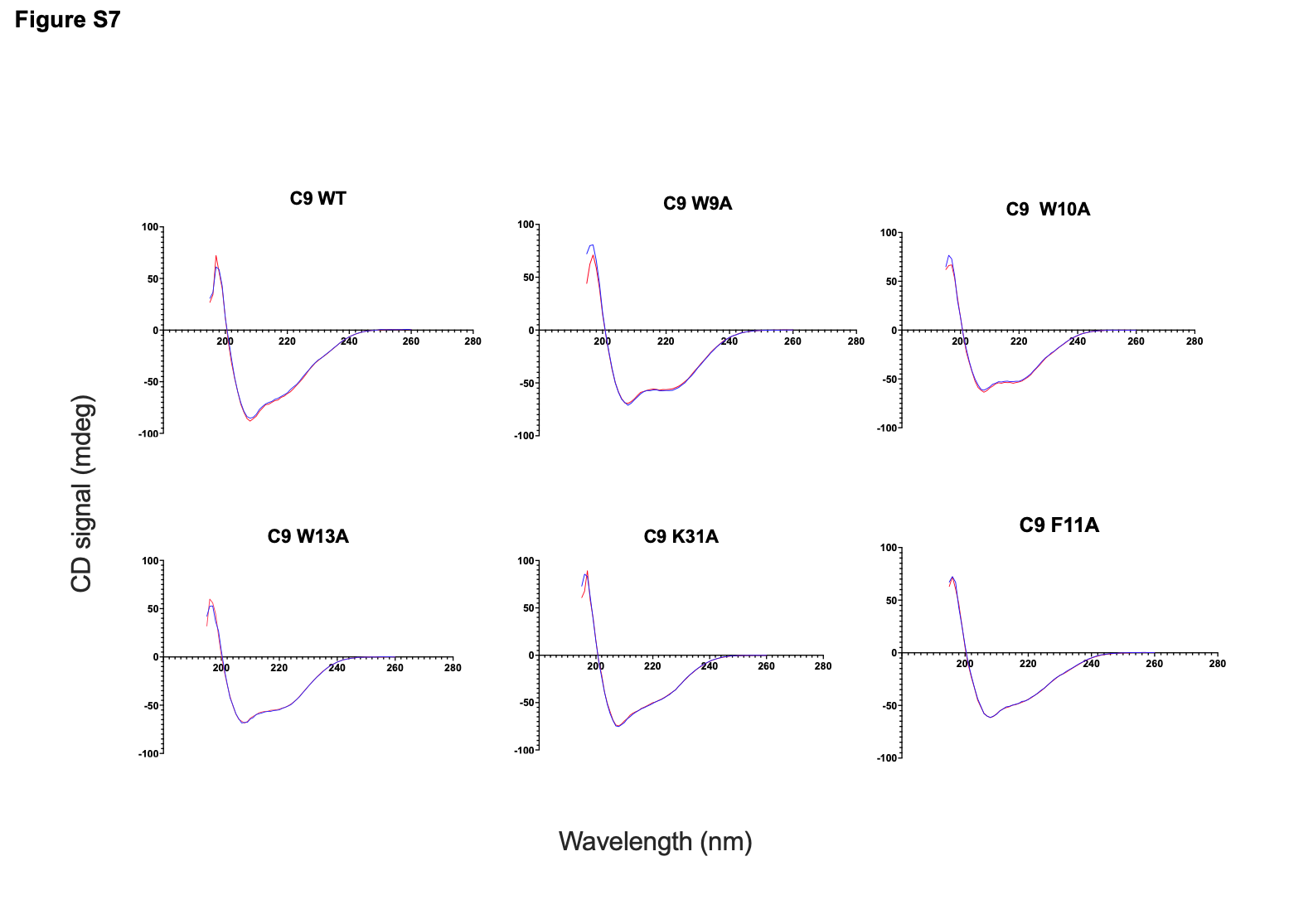
**

**Figure S9.** **Secondary structure analysis of alanine mutated variants of clone C9 using circular dichroism spectroscopy.**

The five alanine mutated variants of C9 that were most affected for their binding to hCEA were analyzed by circular dichroism spectroscopy to monitor their secondary structure contents, compared to the parental C9 affibody. Proteins were analyzed as affibody-His_6_ fusion proteins. The results showed that all six analyzed proteins showed comparable secondary structure contents (mdeg levels), and characteristic minima at 212 and 221 nm for proteins with a large relative content of alpha helices.

**
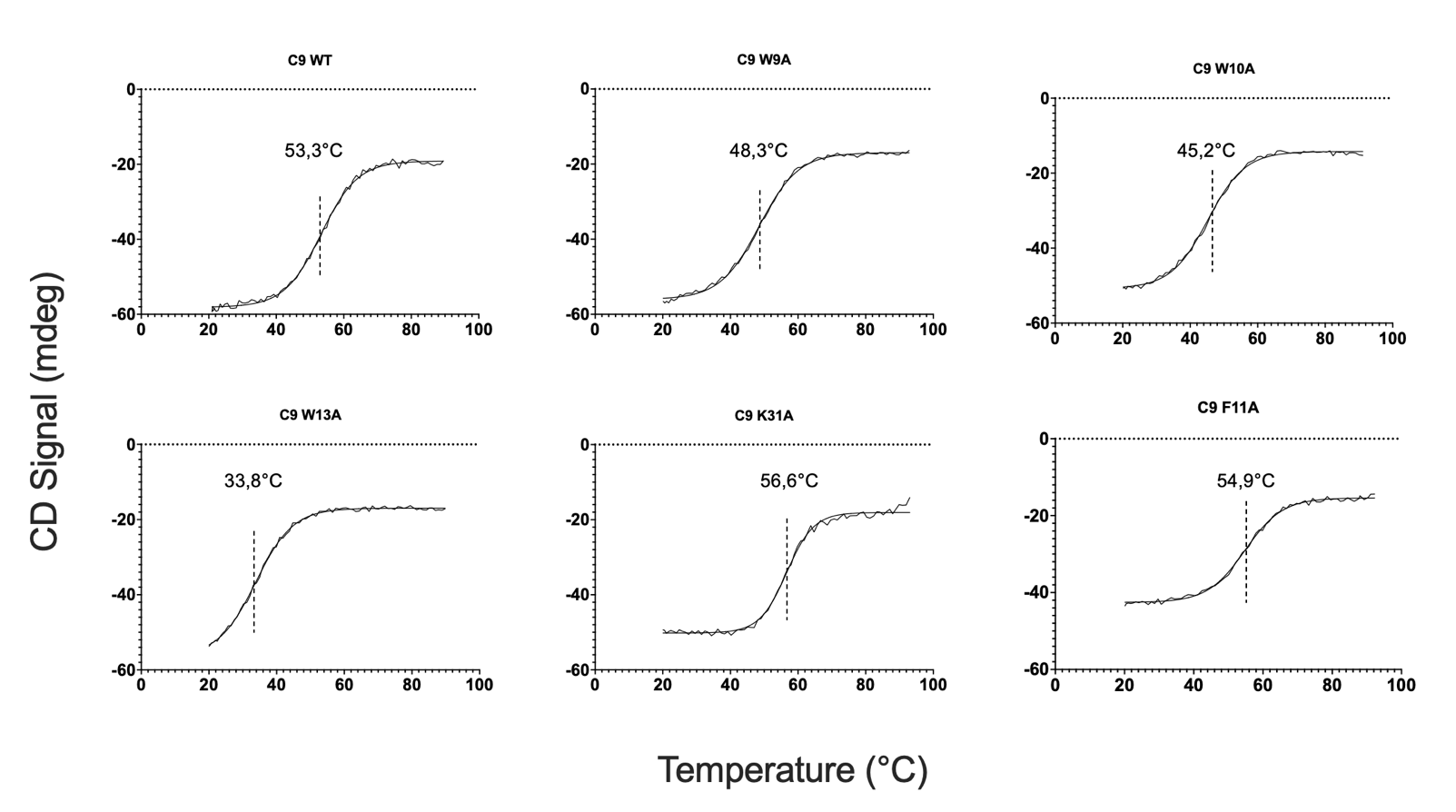
**

**Figure S10. Circular dichroism thermal melting point analyses indicate that single alanine mutations significantly affect the overall stability of different C9 variants.**

The five alanine mutated variants of C9 that were most affected in their binding to CEA were also analyzed by circular dichroism (CD) spectroscopy to monitor their thermal melting points, compared to the parental C9 affibody. Proteins were analyzed as affibody-His_6_ fusion proteins. The two variants K31A and F11A had higher T_m_ values compared to parental C9, while W9A and W10A had reduced Tm values, reflecting lower overall stability. The clone W13A was most affected by the alanine substitution, with a decreased T_m_ value of 19.5°C compared to parental C9.


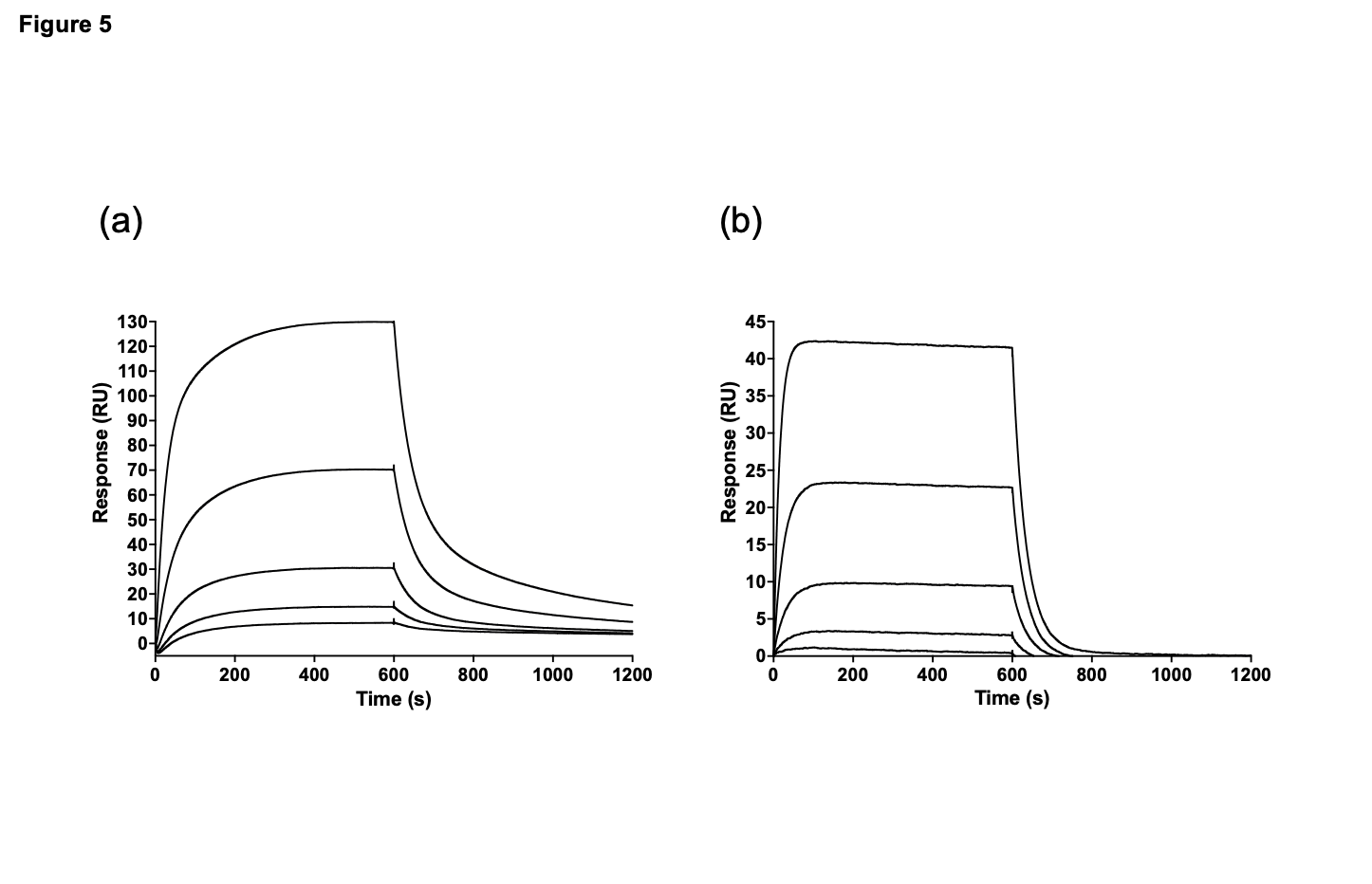


**Figure S11. Binding of C9 to CEA is independent of glycosylation .** Untreated **(a)** or PNGase F-treated **(b)** biotinylated CEA was captured on separate streptavidin containing sensor chip surfaces. Injection of a concentration series of C9-His_6_ protein over both surfaces demonstrated that C9 recognized both forms of CEA. The curve fitting was improved for PNGase F-treated samples indicating a more homogenous ligand on the chip.

**
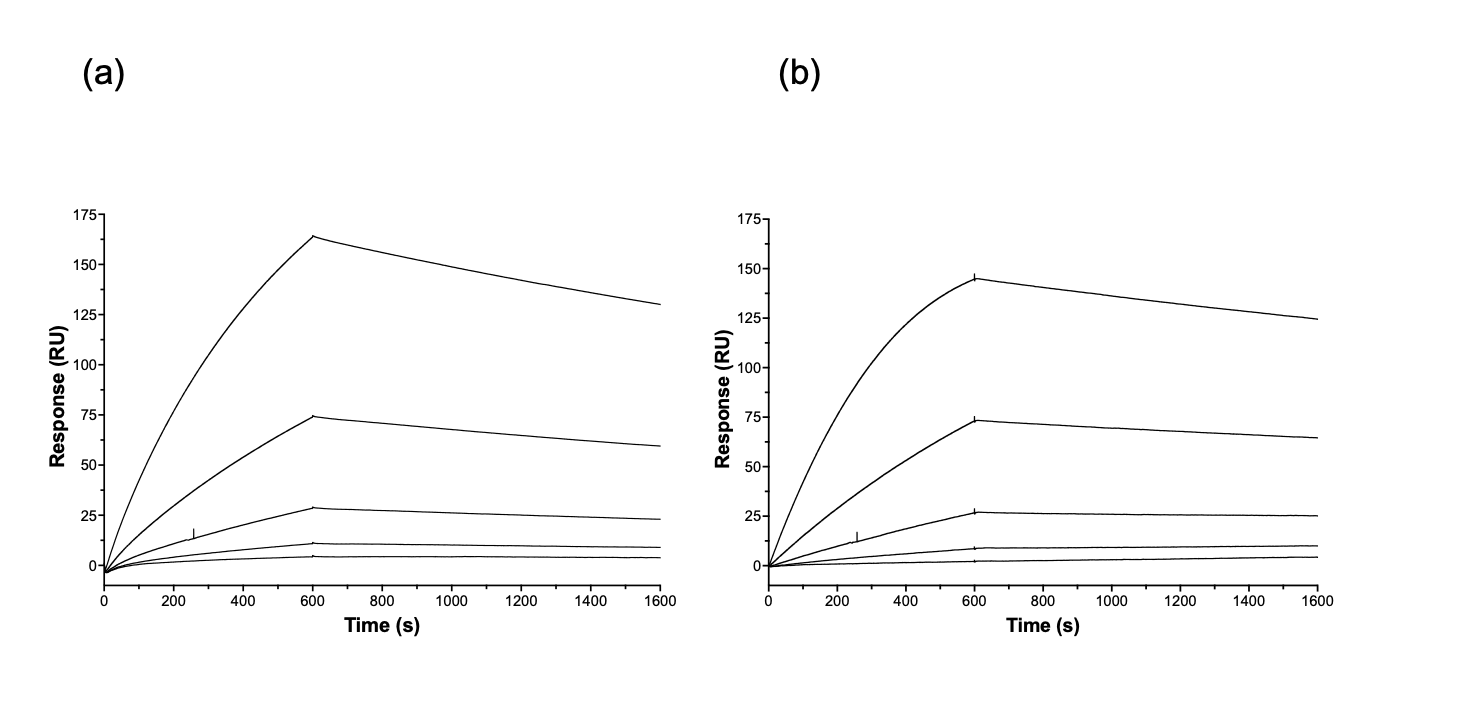
**

**Figure S12. The binding of scFv MFE-23 to CEA is not affected by PNGase F treatment.**

A series of concentrations of the MFE-23 scFv ranging 1-200 nM was injected over either un-treated (a) or PNGase F-treated hCEA and the responses recorded. The analysis showed that the MFE-23 binding profiles were very similar to the differently treated hCEA samples, and thus indicate that the MFE-23 scFv epitope on hCEA does not involve N-linked carbohydrates.

**
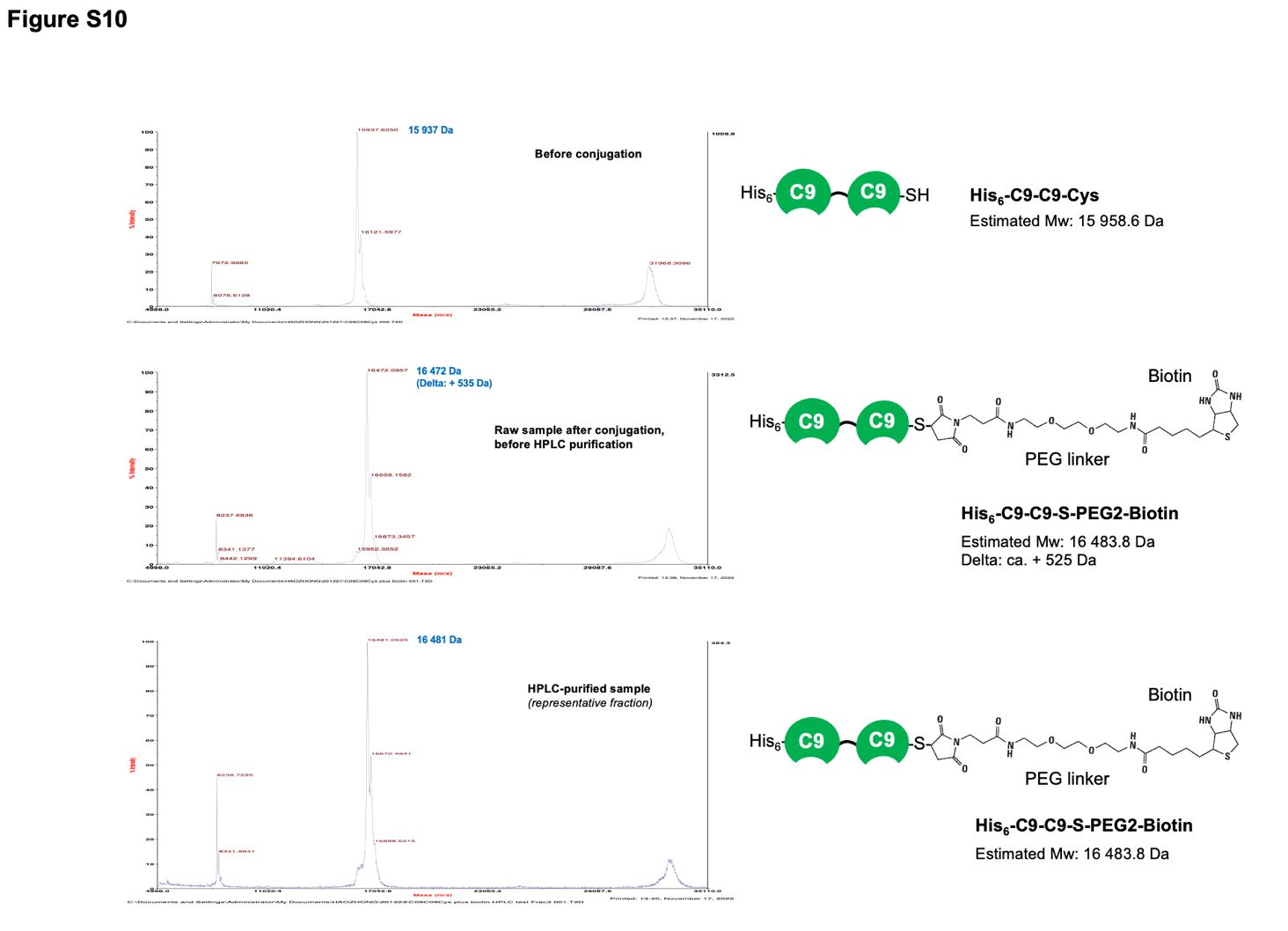
**

**Figure S13.** **High performance liquid chromatography and mass-spectrometry analyses of the His_6_-C9-C9-PEG-Biotin conjugate used in the immunohistochemistry assays reveal high purity.**

High performance liquid chromatography (HPLC) was used to analyze and purify the His_6_-C9-C9-PEG-Biotin conjugates used in the IHC experiments. **Top panel:** Crude His_6_-C9-C9-Cys protein after IMAC purification. **Middle panel:** Raw sample after conjugation between His_6_-C9-C9-Cys and the maleimide-PEG2-Biotin conjugate. **Bottom panel:** Post-purification analysis of HPLC-purified His_6_-C9-C9-PEG-Biotin conjugate. These results showed that the conjugation was efficient, giving a mass increase of ca. 535 Da (close to the expected value), providing highly pure and homogenous product for immunohistochemistry. Molecular masses were determined with MALDI (TOF/TOF) on a Sciex instrument of medium accuracy and resolution.

**
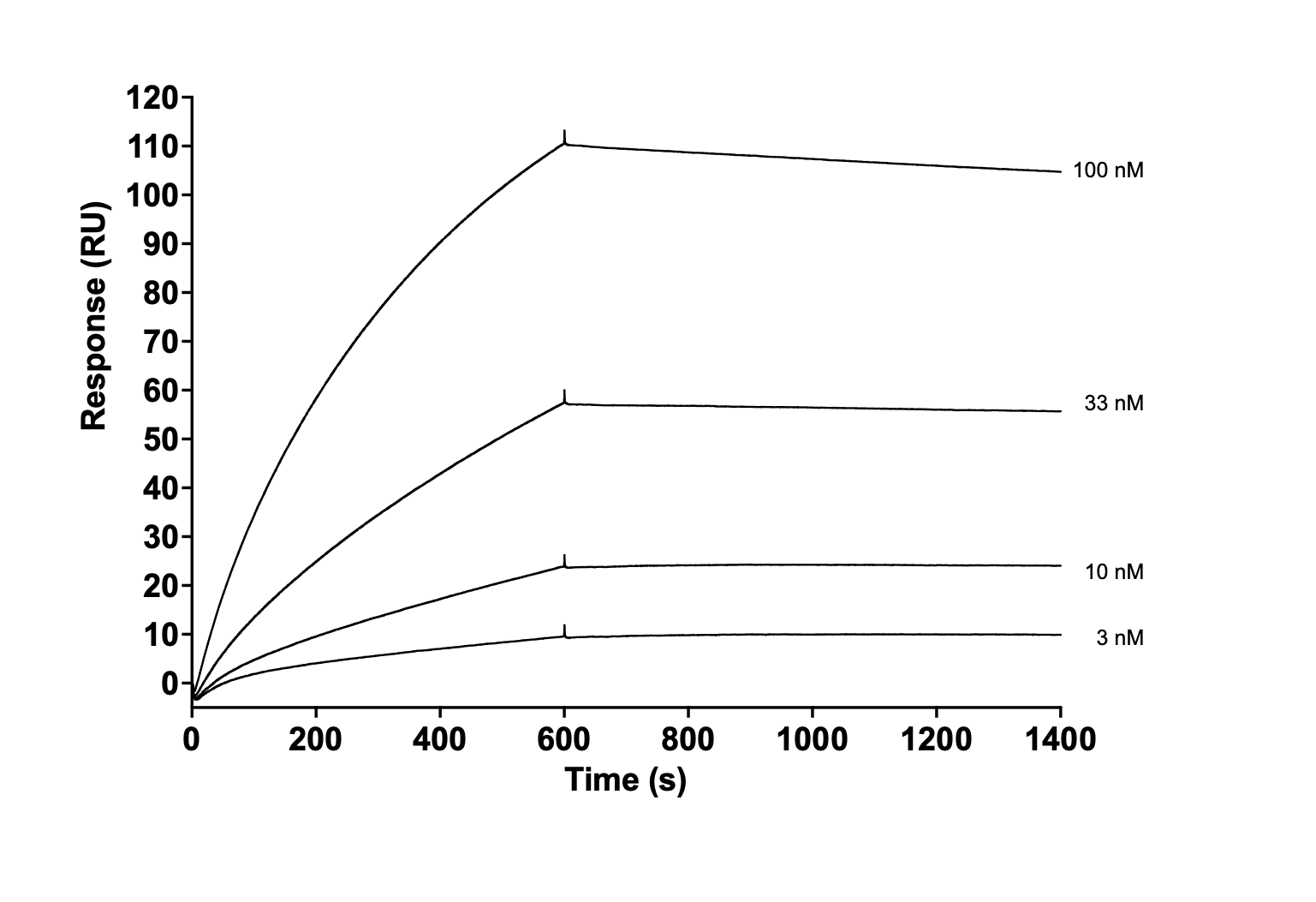
**

**Figure S14.** **Biosensor analysis of the His_6_-C9-C9-PEG-Biotin conjugate.**

The ability of the purified His_6_-C9-C9-PEG-Biotin conjugate to bind to hCEA was assessed using surface plasmon resonance. A concentration series (3-100 nM) was injected over a CEA-coated sensor chip surface. The apparent affinity for CEA was calculated to ca. 1.8 nM, using a 1:1 Langmuir binding model. This value of the apparent affinity was well in line with values obtained for unconjugated C9-C9-His_6_ protein, indicating that the conjugation and HPLC steps had not had any negative effect on the CEA-binding functionality.

**
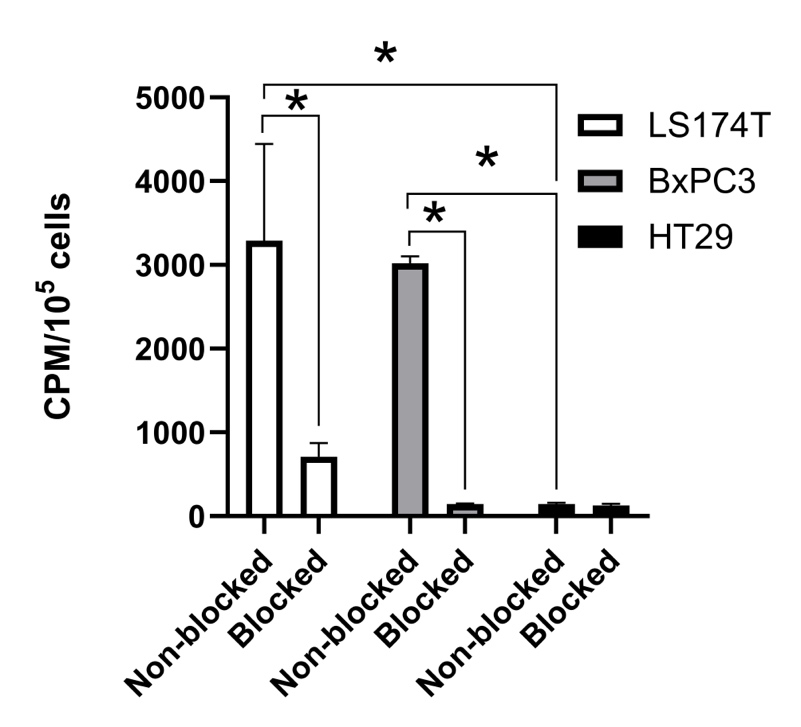
**

**Figure S15. Specificity of ^99m^Tc-labelled C9-C9 binding.** Binding of [^99m^Tc]Tc-C9-C9 to CEA positive LS174T and BxPC3 cells *in vitro*. Blocking of binding sites was obtained using a 100-fold excess of unlabeled C9-C9 protein. CEA-negative HT-29 cells were used as a negative control. The data are presented as an average value from 3 samples ± SD. Asterisks mark significant differences in cell-bound activity (p <0.05, unpaired t-test).

**
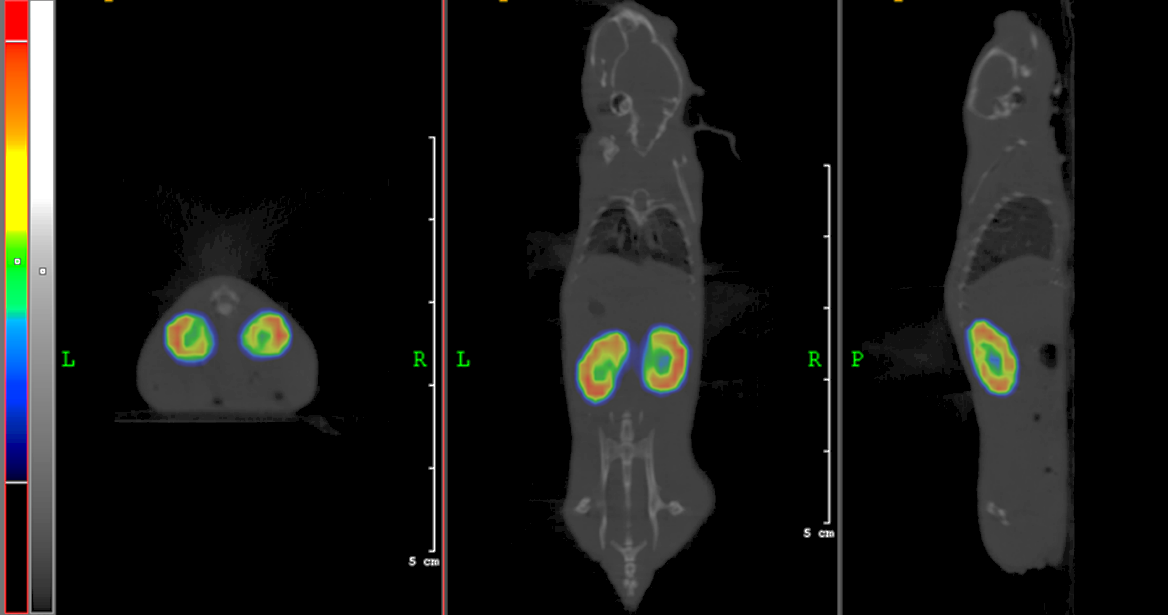
**

**Figure S16. Uptake of [^99m^Tc]Tc-C9-C9-EYEC in renal cortex of mice.** Imaging was performed 4 h after injection of [^99m^Tc]Tc-C9-C9-EYEC. Transversal, coronal and sagittal projections.
